# Combination of Walnut Peptide and Casein Peptide alleviates anxiety and improves memory in anxiety mices

**DOI:** 10.3389/fnut.2023.1273531

**Published:** 2023-10-06

**Authors:** Qinxi Li, Xiuzhen Jia, Qixing Zhong, Zhihui Zhong, Yu Wang, Cheng Tang, Bangcheng Zhao, Haotian Feng, Jingyu Hao, Zifu Zhao, Jian He, Yingqian Zhang

**Affiliations:** ^1^Laboratory of Nonhuman Primate Disease Modeling Research, Department of Neurology, West China Hospital, Sichuan University, Chengdu, China; ^2^State Key Laboratory of Respiratory Health and Multimorbidity, West China Hospital, Sichuan University, Chengdu, China; ^3^Inner Mongolia Dairy Technology Research Institute Co. Ltd., Hohhot, China; ^4^Yili Innovation Center, Inner Mongolia Yili Industrial Group Co., Ltd., Hohhot, China

**Keywords:** walnut peptide, casein peptide, anxiety, memory-improving, serotonin, neurotrophic factor

## Abstract

**Introduction:**

Anxiety disorders continue to prevail as the most prevalent cluster of mental disorders following the COVID-19 pandemic, exhibiting substantial detrimental effects on individuals’ overall well-being and functioning. Even after a search spanning over a decade for novel anxiolytic compounds, none have been approved, resulting in the current anxiolytic medications being effective only for a specific subset of patients. Consequently, researchers are investigating everyday nutrients as potential alternatives to conventional medicines. Our prior study analyzed the antianxiety and memory-enhancing properties of the combination of Walnut Peptide (WP) and Casein Peptide (CP) in zebrafish.

**Methods and Results:**

Based on this work, our current research further validates their effects in mice models exhibiting elevated anxiety levels through a combination of gavage oral administration. Our results demonstrated that at 170 + 300 mg human dose, the WP + CP combination significantly improved performances in relevant behavioral assessments related to anxiety and memory. Furthermore, our analysis revealed that the combination restores neurotransmitter dysfunction observed while monitoring Serotonin, gamma-aminobutyric acid (GABA), dopamine (DA), and acetylcholine (ACh) levels. This supplementation also elevated the expression of brain-derived neurotrophic factor mRNA, indicating protective effects against the neurological stresses of anxiety. Additionally, there were strong correlations among behavioral indicators, BDNF (brain-derived neurotrophic factor), and numerous neurotransmitters.

**Conclusion:**

Hence, our findings propose that the WP + CP combination holds promise as a treatment for anxiety disorder. Besides, supplementary applications are feasible when produced as powdered dietary supplements or added to common foods like powder, yogurt, or milk.

## 1. Introduction

Anxiety disorders are the most common category of mental disorders characterized by feelings of unease, worry, fear, tension, and apprehension (1). According to the World Health Organization (WHO), approximately 380 million individuals worldwide are affected by anxiety disorders (2). It is associated with a decreased quality of life and overall functioning. Besides emotional symptoms, anxiety leads to brain dysfunction, such as depression and dementia (3). Research has found that chronic stress can lead to alterations in both the neuroendocrine and neurotransmitter systems, which in turn impact the creation and management of memories (4, 5). In a 2015 study, Luiz Pessoa explored the multiple interactions between anxiety and cognition functions within the brain. In particular, he looked at how these interactions in the prefrontal cortex (PFC) can minimize response conflicts and selectively affect working memory. Additionally, negative emotions have been shown to influence processes related to memory disproportionately (6).

Although anxiolytic drugs such as antidepressants and Benzodiazepines have been developed to address anxiety symptoms, their efficacy differs among individuals and may lead to addiction or other adverse effects (7). It is worth noting that the Food and Drug Administration has released no new anxiolytic agents since 2007 (8). In addition to drugs, there is growing interest in the potential anxiety-alleviating properties of food like yogurt combined with specific ingredients. This natural, secure, uncomplicated, and budget-friendly approach can be conveniently managed by individuals. Consequently, utilizing food to alleviate anxiety presents a strategy that mitigates the risks linked to psychotropic medications.

Walnut peptide (WP) is a bioactive peptide extracted from walnut protein, a nutritious food rich in polyunsaturated fatty acids, proteins, and minerals (9). WP has been reported to enhance sleep quality memory and cognition in mouse models and human clinical trials (10). Meanwhile, in numerous preclinical and clinical investigations, casein peptide (CP), an essential bioactive peptide derived from milk, has exhibited anxiety-reducing properties, establishing its potential as a therapeutic intervention (11). Due to WP**’**s and CP**’**s nutritional benefits and functional properties, they are widely used in food and beverage products. Adding WP and CP to powder, yogurt, or milk, the dairy products people consume daily is a convenient way to intake various nutrients. Combining WP and CP as the nutrient combination could show antianxiety and memory-improving effects as WP and CP-only exhibits and reduce production costs. WP and CP may relieve anxiety through neurotransmitters, such as dopamine (DA), serotonin, gamma-aminobutyric acid (GABA), and acetylcholine (ACh), which regulate emotions, cognitive functions, and memory formation (12). However, the roles of walnut peptide and casein peptide in neurotransmission remain unclear. Our previous study discovered that the nutrient combination WP + CP at 56.7 + 100 μg/mL showed effects of antianxiety, antioxidants, neuroprotection, and memory improvement in zebrafishes (13), whether they exhibit synergistic effect and alleviates anxiety in anxiety mice model is currently unknown.

This study aims to investigate and highlight the antianxiety and memory-improving effects of WP + CP as a cost-effective nutrient combination in mice. Furthermore, we seek to elucidate the underlying mechanisms involved, including neurotransmitters, neurotrophic factors (BDNF), and microglial cells. We established the chronic anxiety model in mice by elevated open platform. Then, we tested the antianxiety and memory-improving effects of the WP + CP combination and the combination added in powder, yogurt, or milk. Still, we could determine whether the effects of WP and CP combinations are consistent in powder, yogurt, and milk consumed by anxiety mice. This was the first time that the effects of WP + CP combinations were evaluated on anxiety-relieving and memory improvement in rodents.

## 2. Materials and methods

### 2.1. Animals

A total of 100 male C57BL/6 J mice (6–8 weeks old) were acquired from Charles River Laboratories Animal Technology Co., Ltd. (Beijing, China) for this study. The mice were housed in standard cages under specific pathogen-free (SPF) conditions, with a consistent 12-h light–dark cycle, and given standard laboratory chow and distilled water (Manufacturer: Beijing Keao Xieli Feed Co., Ltd., Production License: SCXK (jing) 2015–0013, Batch Number: 20229811). Kangcheng Biotech, Ltd., Co. (Sichuan, China) facilities were used to maintain the mice in a room with ambient temperature (21°C–24°C) and humidity (40%–60%). In each cage, a group of four mice is accommodated. The animal production license number was SYXK (Chuan) 2019-215. Four mice were housed in each cage. All procedures followed the guidelines of the Association for Assessment and Accreditation of Laboratory Animal Care (AAALAC) and were approved by the Institutional Animal Care and Use Committee (IACUC) of the West China Hospital, Sichuan University (Approval No. 2019194A).

### 2.2. Model establishment and grouping

**Figure 1:**
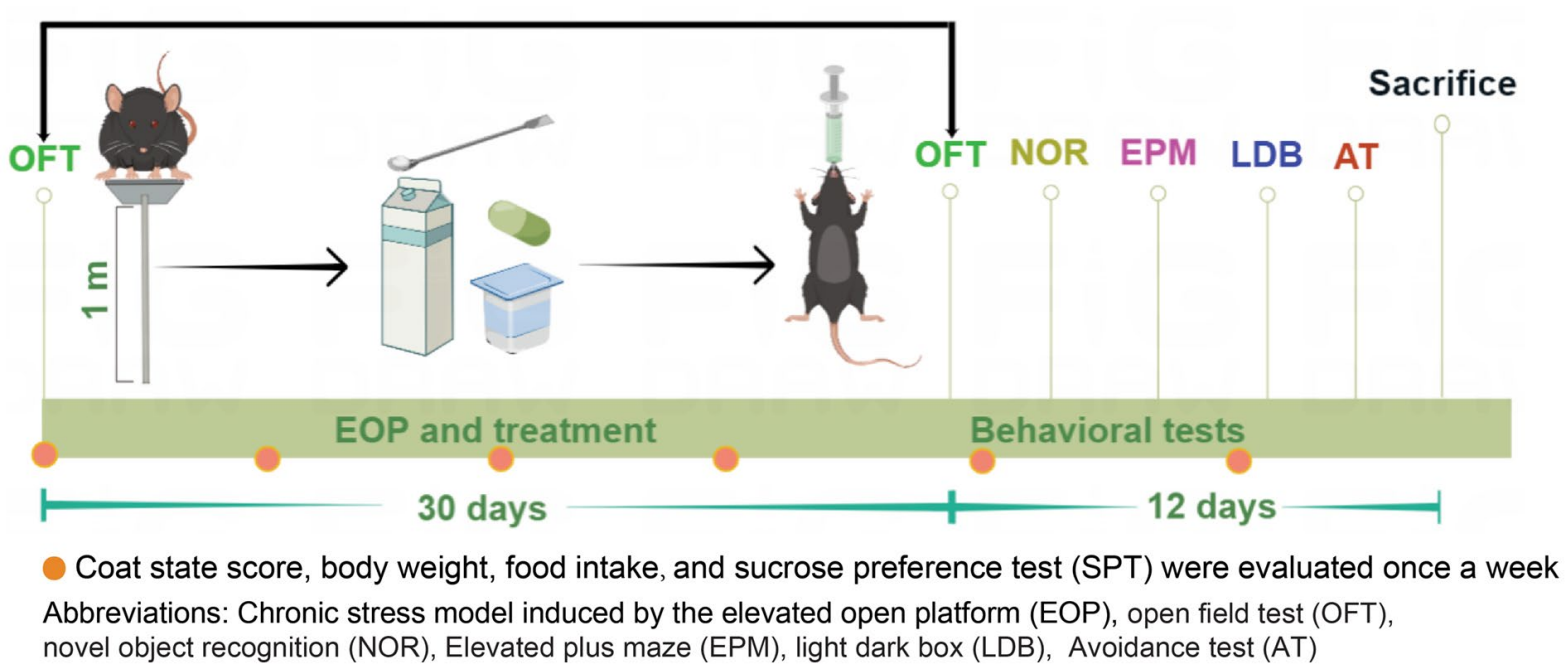
Experimental design and timeline. The experimental design for EOP and the subsequent treatment was illustrated in a timeline extending over 30 days. The mice were subjected to adaptive feeding for 7 days, after which they were randomly assigned to 10 groups based on body weight and total distance traveled. Each group comprised 10 mice. Following the assignment of groups, the mice were subjected to a daily, one-hour session on an elevated platform. They were orally administered with nutrients before being returned to their cages. Behavioral tests (OFT, NOR, EMP, LDB, and AT) were performed after 30 days of model administration to assess anxiety-relieving and cognitive improvement. Additionally, parameters such as body weight, food intake, coat state scores, and SPT were measured every week (as indicated by red dots in the figure). Finally, on day 42 of the experiment, the mice were humanely euthanized, and their brain tissues and serum were collected for analysis.

The anxiety model was established by the elevated open platform (EOP) with a slight adjustment based on a previous study (14). Mice were exposed to the clear square Plexiglas board (10 cm × 10 cm) at 1 m, 1 h per day for 30 days (Figure 1; Supplementary Figure 1). The control mice were not exposed to the elevated open platform (EOP) modeling, yet their living conditions and environment were consistent with those of the other experimental groups.

Mice were randomly assigned to one of 10 groups based on their body weight on day one: control, model, buspirone, powder, yogurt, milk, C, C + powder, C + yogurt, and C + milk. Control, model, and buspirone were designed to test the stability of our modeling system. C was short for the WP + CP combination. Since the combination will enter the powder, yogurt, or milk market, we set the groups of products containing C as C + powder, C + yogurt, and C + milk. The base for the product was powder, yogurt, and milk, respectively. All the nutrients were given by intragastric administration. The protocols utilized for administering intragastric (IG) treatment to mice subjected to the EOP model are described herein (Figure 1). Mice arriving at the laboratory were designated as “eligible mice” if they weighed between 21–25 g and covered 3,000–5,000 cm in the open field test (OFT; Supplementary Figure 2). This criterion ensured a consistent and standardized selection process. Locomotor activity, assessed by distance in the open field test, served as a prerequisite for inclusion in the study and minimized potential confounding factors affecting the reliability and validity of outcomes. Behavioral assessments were conducted from day 30 through day 41, after which the mice from each group were humanely sacrificed and sampled a week following the conclusion of the behavioral experiment.

### 2.3. Sample information

Buspirone hydrochloride (Sigma Chemical Company, St. Louis, MO, United States), a 5-HT1A receptor agonist, was dissolved in saline and given intraperitoneally (IP) once daily for 30 days at 2 mg/kg, as previously reported (15). The powder, yogurt, and milk doses were determined according to the recommended human dose (16–18). The information of combination and products containing combination are shown in Table 1.

**Table 1:** The information of samples tested.

Group	Name	Origin	Human dose	Mice dose	Calculation procedure
Positive drug	Buspirone Hydrochloride	Sigma Chemical Co	20–40 mg	2 mg/kg	
Base	Powder	Inner Mongolia Yili Industrial Group Co., Ltd	25 g/d	3791.67 mg/d·kg	25 g/d ÷ 60 kg × 1,000 mg/g = 416.67 mg/d·kg × 9.1 = 3791.67 mg/d·kg
	Yogurt		200 mL/d	30.33 mL/d·kg	200 mL/d ÷ 60 kg × 9.1 = 30.33 mL/d·kg
	Milk		200 mL/d		
Combinations	C	WP: The plant extract, a bioactive peptide extracted from the protein of walnut residues (Sinphar Group Co.) CP: Milk (Guangdong Huapeptides Biotechnology Co., Ltd.)	170 + 300 mg	25.87 + 45.50 mg/kg	WP: 170 mg/d ÷ 60 kg = 2.83 mg/d·kg (Human dose) × 9.1 = 25.75 mg/d·kg (Mice dose) CP: 300 mg/d ÷ 60 kg = 5 mg/d·kg (Human dose) × 9.1 = 45.50 mL/d·kg (Mice dose)
Products	C + powder	Inner Mongolia Yili Industrial Group Co., Ltd	Powder 25 g/d, containing combinations 170 + 300 mg	3791.67 mg/kg	25 g/d ÷ 60 kg × 1,000 mg/g = 416.67 mg/d·kg (Human dose) × 9.1 = 3791.67 mg/d·kg
	C + yogurt		Yogurt 200 mL/d, containing combinations170 + 300 mg	30.33 mg/kg	200 mL/d ÷ 60 kg × 9.1 = 30.33 mL/d·kg (The products were ready to administer, requiring no preparation.)
	C + milk		Milk 200 mL/d, containing combinations 170 + 300 mg	30.33 mg/kg	

The general packaging size for liquid dairy products is 200 mL; the powder is around 25 g dissolved in 180–200 mL water. The administration dosage for the mice was converted according to the surface area of a 60 kg human body. According to the conversion ratio between humans and mice (9.1) (19), the daily combination for mice can be calculated as the following formula: conversion ratio of surface area between mice and humans (9.1) × a daily dose of an adult (25 g/d powder)/average weight of adults (60 kg), that is 9.1 × 25 g/60 kg = 3.79 g/kg. The feeding volume was 20 mL/kg for each mouse. The combinations of WP + CP were tested in three dosages: 85 + 200 mg, 170 + 300 mg, and 170 + 600 mg. The open field test (OFT) and elevated-plus maze (EPM) showed that a combination with 170 + 300 mg had the best antianxiety-improving effects (Supplementary Figures 3A–C). Furthermore, there were discernible outcomes observed in the light–dark box (LDB), novel object recognition (NOR), and passive avoidance test (PAT) tests for both the C-medium and C-high groups (Supplementary Figures 3D–F). Therefore, the medium dosage (170 mg WP + 300 mg CP) was selected for the following studies.

### 2.4. Assessment of animals’ physical states

Body weight, food intake, and coat state score were evaluated every Wednesday between 10 and 12 a.m. (Figure 1). More details were shown in Supplementary material.

### 2.5. Behavioral assessment

#### 2.5.1. Open-field test

Each mouse was carefully placed in the center of an open field box (50 × 50 × 50 cm) and allowed to explore for 10 min, following established procedures (20). The software was used to record and analyze the distance traveled within the open field and the times each mouse entered the central area of the arena (measuring 25 × 25 cm). The Topscan Package provided by Clever Sys Inc. based in the United States was used to quantify these parameters accurately.

#### 2.5.2. Elevated plus-maze test

The Elevated plus-maze (EPM) consisted of two arms, open and closed, with the closed arms having 20 cm high walls. Both arms were 30 cm in length and 5 cm in width, and the EPM was elevated to a height of 60 cm above the ground, forming a 90° angle between the arms. Before the test, the mice could explore the central area for 5 min while facing the open arm. The open arm entry percentage (OE%) was calculated by dividing the number of entries into the open arm by the total number of entries into both arms and multiplying by 100. The open arm time percentage (OT%) was calculated by dividing the time spent in the open arm by 300 s (the total test duration) and multiplying by 100 (21).

#### 2.5.3. Light–dark box test

As previously mentioned, anxiety was tested in the light/dark box (LDB) (22). A Plexiglas box with two equal chambers (18 × 12 × 12 cm) and an opening in the middle (5 cm length × 5 cm width) was used. The experiment was conducted in a dimly lit room (60 lx), and an infrared camera recorded the dark box. The mice were placed in the lightbox facing the dark box, and their exploration behaviors were recorded for 10 min after they crossed the opening (22).

#### 2.5.4. Novel object recognition test

Recognition was assessed using the Novel object recognition (NOR) test, which comprised a learning trial and a subsequent test trial (23). In the former, two identical objects were presented to the mouse, which was allowed to explore them for 10 min in an open field box (60 × 25 × 25 cm). After a one-hour interval, the mouse underwent a test trial, during which one of the objects was replaced with a novel object. The mouse was given 5 min to explore both objects. The discrimination ratio of the recognition index was calculated using a specific formula (23).


$$\mathrm{Discrimination}\;\mathrm{ratio}=\frac{\begin{array}{l} \mathrm{Ratio}\;\mathrm{of}\;\mathrm{total}\;\mathrm{time}\;\mathrm{spent}\;\\ \mathrm{with}\;\mathrm{the}\;\mathrm{novel}\;\mathrm{object}\;\mathrm{divided} \end{array}}{\begin{array}{l} \mathrm{Total}\;\mathrm{time}\;\mathrm{spent}\;\mathrm{on}\;\mathrm{exploring}\;\\ \mathrm{either}\;\mathrm{object}\;\mathrm{multiplied} \end{array}}\times 100\%$$

#### 2.5.5. Avoidance test

The Avoidance test (AT) protocol was conducted in a light/dark shuttle box with minor adjustments from day 36 to day 41, as previously described (24). The AT, including the active avoidance test (AAT) and passive avoidance test (PAT), involved three consecutive days, starting with an adaptation day where the mouse explored both compartments for 2 min. On the training day, the mouse received an electric shock upon entering the dark compartment, while on the PAT test day, the mouse explored the bright compartment without shock. The fourth day was designated the AAT, where the mouse explored the dark compartment without shock.

### 2.6. Sucrose preference test

To prepare for the sucrose preference test (SPT), a 1% sucrose solution and distilled water were simultaneously introduced into each mouse’s cage. This allowed for 2 days of taste adaptation. On the third day, after a six-hour fasting and water deprivation period, the mice were placed individually in cages that contained pre-weighed bottles of 1% sucrose solution and distilled water. Six hours later, the positions of these bottles were swapped. Following an additional 12 h, both bottles were removed. The remaining liquid was measured to calculate the sucrose preference of the mice using the following formula (25):


$$\mathrm{Sucrose}\;\mathrm{preference}\;\mathrm{percentage}=\frac{\mathrm{Sucrose}\;\mathrm{consumption}}{\mathrm{Total}\;\mathrm{liquid}\;\mathrm{consumption}}\times 100\%$$

SPT was performed weekly, consistent with the time points of body weight, food intake, and coat state scores (Figure 1).

### 2.7. Serum corticosterone and adrenocorticotropic hormone assay

After blood collection between 7:00–8:00 a.m., mice were euthanized by carbon dioxide inhalation. Serum concentrations of corticosterone and adrenocorticotropic hormone (ACTH) were determined using an enzyme-linked immunosorbent assay (ELISA) kit from TECAN, Germany (RE52211) and Abcam, United Kingdom (ab263880), respectively.

### 2.8. Liquid chromatograph mass spectrometer

The brains of mice were weighed and homogenized in RNase-free water at a 1:4 ratio for detection. The samples were centrifuged at 12,000 g for 5 min at a temperature of 4°C. Protein precipitation was performed using 50 μL of the brain homogenate, 5 μL of blank, and 200 μL of acetonitrile internal standard. A standard curve was prepared using a 1 mg/mL mother solution diluted with 50% acetonitrile. Standard curve and biological samples ranging from 50~2,000 ng/mL were prepared. Quality control samples were also prepared at 5, 10, 50, 800, and 1,600 ng/mL. The protein precipitator containing IS was added to each standard working solution, QC working solution, and unknown concentration samples, which were then vortexed for 30 s. After centrifugation, the supernatant was diluted with water. Brain extracts were injected into the Analyst® System, and the data are expressed as ng per g tissue. HPLC-MS, using Analyst® software, detected serotonin, ACh, GABA, and DA in the prefrontal cortex (PFC). A Waters column was utilized with mobile phases A and B. The chromatographic gradient is presented in Table 2, and the injection volume was set at 5 μL, with dexamethasone and verapamil as internal standards.

**Table 2:** The chromatographic gradient of mobile phase (A: water, and B, acetonitrile).

Time (min)	Flow rate (mL/min)	A (%)	B (%)
Initial	0.6	90	10.0
0.30	0.6	90	10.0
2.00	0.6	10.0	90.0
2.50	0.6	10.0	90.0
2.60	0.6	90.0	10.0
3.50	0.6	90.0	10.0

### 2.9. RNA extraction and real-time reverse transcription polymerase chain reaction analysis

The modified guanidine isothiocyanate-phenol-chloroform method, incorporating RNX+ reagent, was used to isolate RNA from the right hemisphere hippocampus, following the manufacturer**’**s protocol, with subsequent treatment using RNase-free water to prevent DNA contamination. Spectrophotometry, using a Thermo-Nano Drop 2000c-spectrophotometer, was used to quantify the concentration and purity of all RNA samples and determine the mean absorbance ratio and optical density (OD) at 260/280 nm. The cDNA was synthesized using HiScript III RT SuperMix for qPCR (+gDNA wiper; Vazyme, R323, China) and ChamQ Universal SYBR qPCR Master Mix (Vazyme, Q711, China). The primers used to analyze β-actin and brain-derived neurotrophic factor (BDNF) were β-actin, 5′-CCACCATGTACCCAGGCATT-3′ (forward), and 5′-CAGCTCAGTAACAGTCCGCC-3′ (reverse); BDNF, 5′-TCCGGGTTGGTATACTGGGTT-3′ (forward) and 5′-GCCTTGTCCGTGGACGTTT-3′ (reverse). RT-PCR analyses were conducted using the Bio-Rad CFX Manager (Bio-Rad, CFX Connect, United States) according to the manufacturer**’**s protocol. The DNA amplification process consisted of an initial cycle at 95°C for 30 s, followed by 40 cycles of denaturation (95°C for 10 s), annealing (60°C for 30 s), and extension (95°C for 15 s). The 2 −ΔΔCt method was used to calculate the expression of β-actin and BDNF, with a single calibrated sample serving as the reference for comparison with the expression of all unknown samples.

### 2.10. Ionized calcium-binding adapter molecule 1 immunohistochemistry

Ionized calcium-binding adapter molecule 1 (Iba1) is a protein that marks microglial cells. The mouse brains were fixed in 10% buffered formalin and then embedded in paraffin. For immunohistochemical analysis, four μm sections were prepared. The anti-Iba1 antibody from Wako (Richmond, VA) was used at a concentration of 1:1,000 to detect Iba1. We quantified the number of Iba1-positive cells, the total number of cells, and the number of positively stained cells in the areas with the highest tumor-cell density in 10 non-overlapping microscopic fields (at 400 × magnification) of tumor-bearing brains taken from mice in each group.

### 2.11. Statistical analysis

The statistical analysis was conducted using SPSS 26.0 software (IBM Ltd., United Kingdom). Data conforming to normal distributions were represented as mean ± standard errors of the mean (SEM). Unpaired *t*-tests were utilized to analyze the differences between the control and model groups. A one-way Analysis of Variance (ANOVA) followed by the *post hoc* Tukey Dunnett**’**s multiple comparison tests was used to analyze differences among the model, buspirone, and each treatment group when the data followed a normal distribution and the variances were equal. A repeated measures ANOVA was employed to examine group variations in body weight, food intake, coat state, sucrose preference, and fecal amount. Interaction effects between repeated indicators and days were tested by Pillai’s trace. In the case of interactions observed between a specific variable and days, the differences among each group were compared at the final time point. If no interactions were present, post-hoc analysis using Bonferroni’s multiple comparison tests was conducted. The correlations between each effect were performed using Pearson**’**s correlation. For all analyses, two-sided *p*-values < 0.05 was considered statistically significant.

## 3. Results

### 3.1. WP and CP combination showed apparent antianxiety effects

#### 3.1.1. Changes of general states in each group

**Figure 2:**
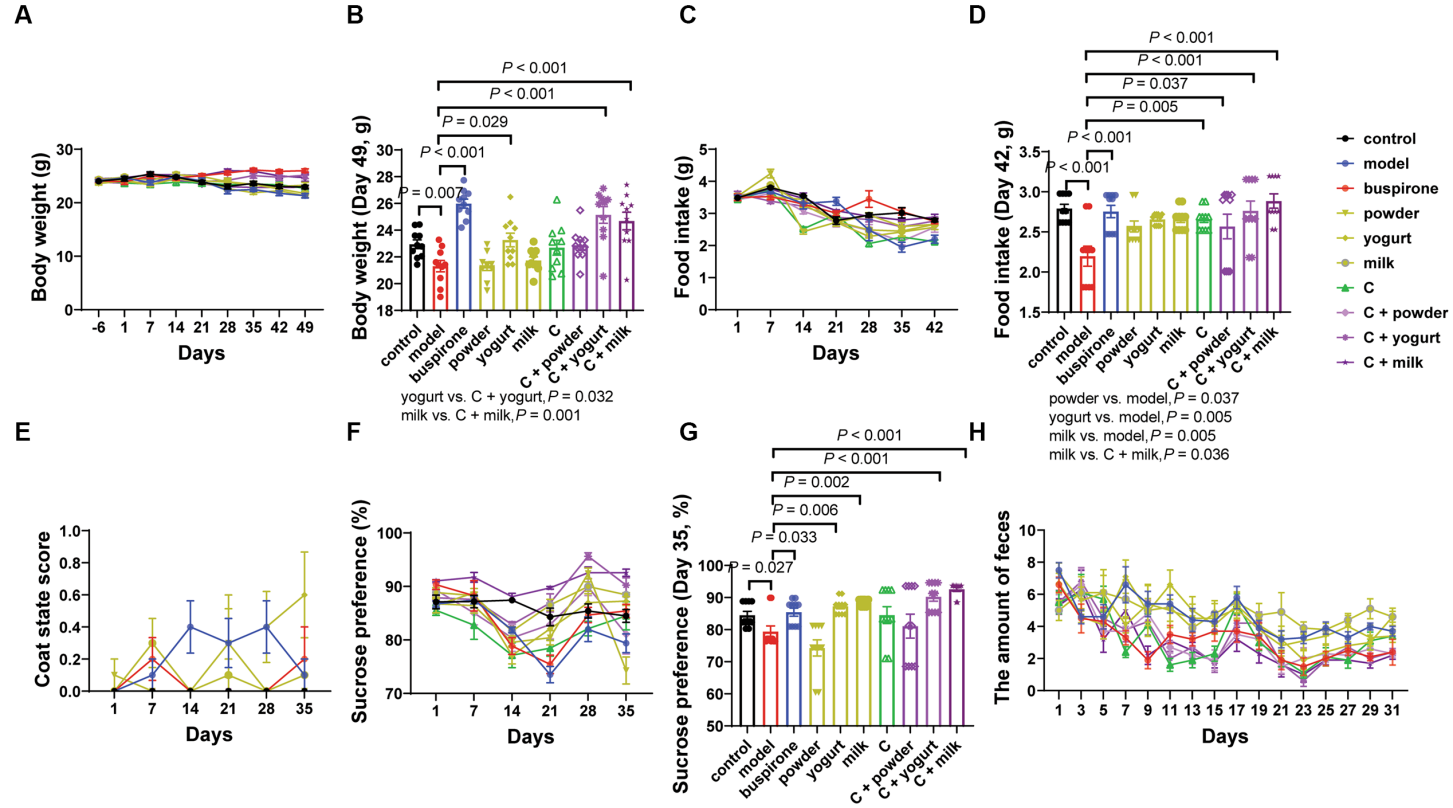
The influence of the combined administration of WP + CP on the fundamental physiological data in mice. Throughout the experiment, changes in body weight **(A)**, and at the experimental endpoint of 49 days, discernible variations in body weight were observed among the different groups of mice **(B)**. Food intake **(C)** was monitored weekly, and at the 42-day experimental endpoint, conspicuous disparities in food consumption were observed across the various mouse cohorts **(D)**. Changes in coat state score **(E)**, SPT **(F)**, and the amount of feces **(H)** were recorded every week. On the 35th day of the experiment, notable variations in the percentage of water preference among the different groups were detected **(G)**. The data, representing mean values ± SEM, *n* = 10 in each group, were analyzed using repeated measures analysis of variance (ANOVA) to investigate potential differences among groups. If the interactions between one index and days existed, we compared the difference among each group of the last time points **(B,D,F)**. If the interactions did not exist, post-hoc analysis (Bonferroni**’**s multiple comparison tests) could be done next.

During the anxiety model establishment and combination administration, the body weight, food intake, coat state score, and sucrose preference test (SPT) were determined weekly (Figure 1). We tested three WP + CP combination dosages, 85 + 200 mg, 170 + 300 mg, and 170 + 600 mg, respectively. During the anxiety experiment, the C-low group demonstrated markedly reduced scores on the Open field test (OFT) and EPM assessments compared to the C-medium and C-high groups. In the cognitive tests, discernible disparities in the NOR and PAT tests were solely observed with the C-medium and C-high groups when contrasted against the control group, thereby substantiating the decision to establish the combination dosage as the medium dose: 170 + 300 mg (Supplementary Figure 3). The body weights were maintained steady, slightly decreasing in some groups after EOP, and there was an interaction effect between body weight and days (Group * day, *F* = 3.027, df = 63, *p* < 0.001; Figure 2A). The simple effect of body weight was shown in Supplementary Tables 1A,B. On day 49, the model group had the lowest body weight compared with the control (*t* = 3.054, df = 18, *p* = 0.007; Figure 2B). One-way ANOVA was employed to analyze the difference between the model group and all the treatment groups (Buspirone, powder, yogurt, milk, C, C + powder, C + yogurt, and C + milk) in the present study. Since differences were evident among these eight groups [*F*_(8, 81)_ = 13.04, *p* < 0.001], a *post hoc* Bonferroni test was conducted, revealing that buspirone, yogurt, C + yogurt, and C + milk led to a tremendous increase in mice’s body weight compared to the model group (*p* < 0.001, *p* = 0.029, *p* < 0.001, *p* < 0.001, respectively; Figure 2B). Compared with yogurt and milk, C + yogurt and C + milk increased body weight (*p* = 0.032, *p* = 0.001; Figure 2B). Besides body weight, the food intake decreased gradually in general, especially in the model group, possibly due to anxiety-induced appetite loss. Due to the interaction effects between food intake and days (Group*day, *F* = 4.906, df = 54, *p* < 0.001; Figure 2C), we only compared changes in food intake on the last time point, Day 42. The simple effect of food intake was analyzed in Supplementary Table 2B. Food intake was the least in the model group, significantly lower than control (*t* = 4.358, df = 18, *p* < 0.001; Figure 2D). Differences existed in the model and treatment groups [*F*_(8, 81)_ = 4.337, *p* = 0.001]. The *post hoc* Bonferroni test revealed that buspirone, C, C + powder, C + yogurt, and C + milk could all increase the food intake of anxiety mice (*p* < 0.001, *p* = 0.005, *p* = 0.037, *p* < 0.001, *p* < 0.001; Figure 2D). The simple effects of food intake were analyzed in Supplementary Tables 2A,B.

The EOP represents a prominent approach to anxiety modeling. Our study evaluated coat condition and anhedonia in mice following 30 days of long-term intragastric therapy and modeling. The interaction effect was found between coat state scores and days (Group*days, *F* = 2.173, df = 45, *p* < 0.001; Supplementary Tables 3A,B), and we could see the highest level of coat state scores in the model group (Figure 2E). SPT was performed six times, and an interaction effect existed between sucrose preferences and days (Group*days, *F* = 5.643, df = 45, *p* < 0.001; Supplementary Tables 4A,B). It showed that the sucrose preference was consistently decreased during the early stages of the modeling process. However, in the later stages (21 days later), almost all groups showed varying degrees of recovery (Figure 2F), and the simple effect of groups. When comparing changes in sucrose preference on day 35, the model was lower than the control (*t* = 2.402, df = 18, *p* = 0.027). One-way ANOVA was employed to analyze the model group and treatment groups in the present study [*F*_(8, 81)_ = 8.492, *p* < 0.001]. Following the analysis, a *post hoc* Bonferroni test was conducted, revealing that buspirone, yogurt, milk, C + yogurt, and C + milk help increase sucrose preference (*p* = 0.033, *p* = 0.006, *p* = 0.002, *p* < 0.001, *p* < 0.001; Figure 2G).

Anxiety mice exhibited lower activity in the open-field experiment and preferred the periphery over the central area. Correspondingly, mice in an anxious state produced more fecal particles (26). Therefore, we aimed to measure mice**’**s anxiety levels by quantifying fecal particle production on an elevated platform. Clearly, there was an interaction effect between feces amount and days (Group*days, *F* = 1.746, df = 120, *p* < 0.001; Supplementary Tables 5A,B). The C + milk group exhibited a lower fecal particle count than the model group. In contrast, the milk group demonstrated significantly higher particle levels than the C + milk group (Figure 2H).

#### 3.1.2. Results of behavioral tests in evaluating anxiety states

**Figure 3:**
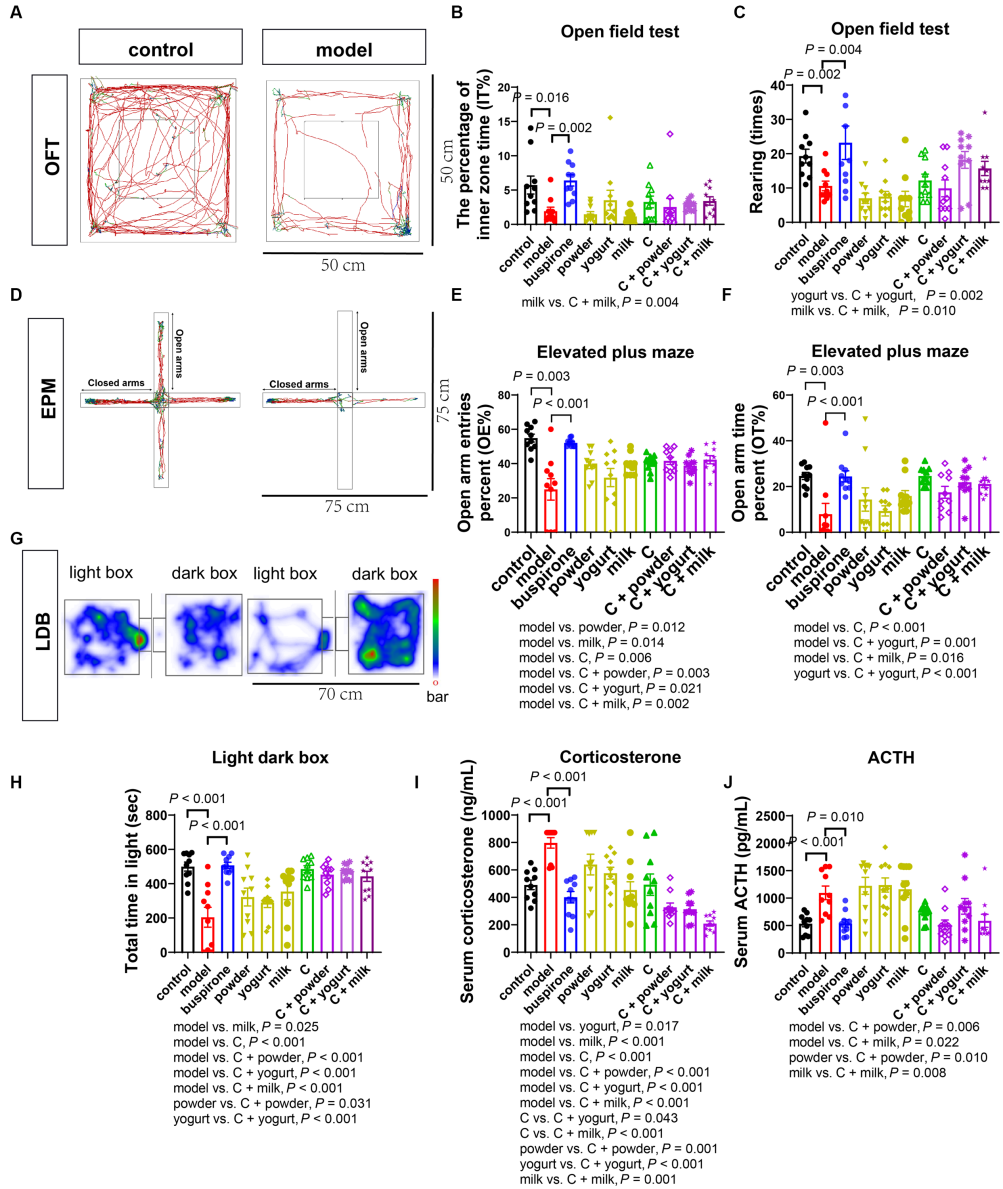
Effects of WP + CP on anxiety relieving in mice. **(A–C)** OFT performed on the 30th day. The representative pictures of control and model **(A)**, changes of the percentage of inner zone time **(B)**, and rearing times **(C)** by OFT in each group. **(D–F)** EPM performed on the 32nd day. Representative pictures of EPM **(D)**, changes of OE% **(E)**, and OT% **(F)** by EPM in each group. **(G)** LDB by trajectories on the 35th day. **(G,H)** LDB performed on the 35^th^ day. Representative pictures of LDB **(G)** and changes of total time in light of each group **(H)**. **(I,J)** Serum concentrations of corticosterone **(I)** and ACTH **(J)** in each group. Data represent mean ± SEM; *n* = 10 in each group.

During days 30–35, a sequential battery of tests was conducted to assess mice’s anxiety-like behaviors, including the OFT, EPM, and LDB. The percentage of time spent in the inner zone was lower in the model group than in the control (*t* = 2.654, df = 18, *p* = 0.016). At the same time, buspirone treatment increased the center**’**s exploration of mice compared to the model (*t* = 2.654, df = 18, *p* = 0.016; Figures 3A,B). The model group exhibited significantly fewer rearing times than the control (*t* = 3.557, df = 18, *p* = 0.002). One-way ANOVA was employed to analyze the model group and treatment groups in the present study [*F*_(8, 81)_ = 5.186, *p* < 0.001]. Following the analysis, a *post hoc* Bonferroni test was conducted, revealing that buspirone, C + yogurt, and C + milk induced more rearing times relative to the yogurt and milk (*p* = 0.004, *p* = 0.002, *p* = 0.010; Figure 3C). Supplementary Table 6 shows cases of the additional metrics of the open field activity.

During the EPM test, the model group exhibited the lowest percentage of open-arm entries and time spent in the open arms. However, treating with buspirone, milk, C, and C + powder/yogurt/milk increased open-arm exploration compared to the model (Figures 3D–F). This suggests that the combination and its products effectively alleviate anxiety-like behavior in mice.

We employed the LDB test as a third behavioral assay to assess the anxiety state. The model group showed a remarkably shorter time spent in the light box than the controls (*t* = 4.632, df = 18, *p* < 0.001). One-way ANOVA was employed to analyze the model group and treatment groups in the present study [*F*_(8, 81)_ = 8.536, *p* < 0.001]. Following the analysis, a *post hoc* Bonferroni test was conducted, revealing that buspirone C, C + powder, C + yogurt, and C + milk prolonged the time spent in the lightbox (all *p-*value < 0.001). In particular, the C + powder and C + yogurt groups exhibited a substantially longer time spent in the light box, suggesting a reduced anxiety-like phenotype (Figures 3G,H). Trace of OFT, EPM, and LDB in the treatment group was shown in Supplementary Figure 4.

#### 3.1.3. Changes in stress-related hormones

We subsequently investigated the alterations in stress-related hormones in the serum, the commonly used biomarkers in clinical settings that reflect changes in the hypothalamic–pituitary–adrenal axis. Notably, the levels of corticosterone and ACTH were elevated in the model compared with the control ([corticosterone]: *t* = 5.927, df = 18, *p* < 0.001; [ACTH]: *t* = 4.119, df = 18, *p* < 0.001, Figures 3I,J). Excluding the control group, a one-way ANOVA analysis was conducted on the remaining groups [*F*_(8, 81)_ = 13.23, *p* < 0.001]. Regarding serum corticosterone levels, group C demonstrated elevated concentrations in comparison to C + yogurt (*p* = 0.043) and C + milk (*p* < 0.001), followed by a subsequent decrease in serum corticosterone upon the introduction of C to the three bases (Figure 3I). One-way ANOVA was employed to analyze the model group and treatment groups in the present study [*F*_(8, 81)_ = 6.446, *p* < 0.001]. As the upstream regulatory factor, changes in serum ACTH were discovered to be more responsive than corticosterone reductions in serum ACTH were observed in C + powder/milk compared to the model (*p* = 0.006, *p* = 0.022). Furthermore, C + powder and C + milk triggered lower serum ACTH expression levels than the two bases ([powder vs. C + powder]: *p* = 0.010; [milk vs. C + milk]: *p* = 0.008; Figure 3J).

### 3.2. The WP + CP combination enhanced memory In mice with anxiety

**Figure 4:**
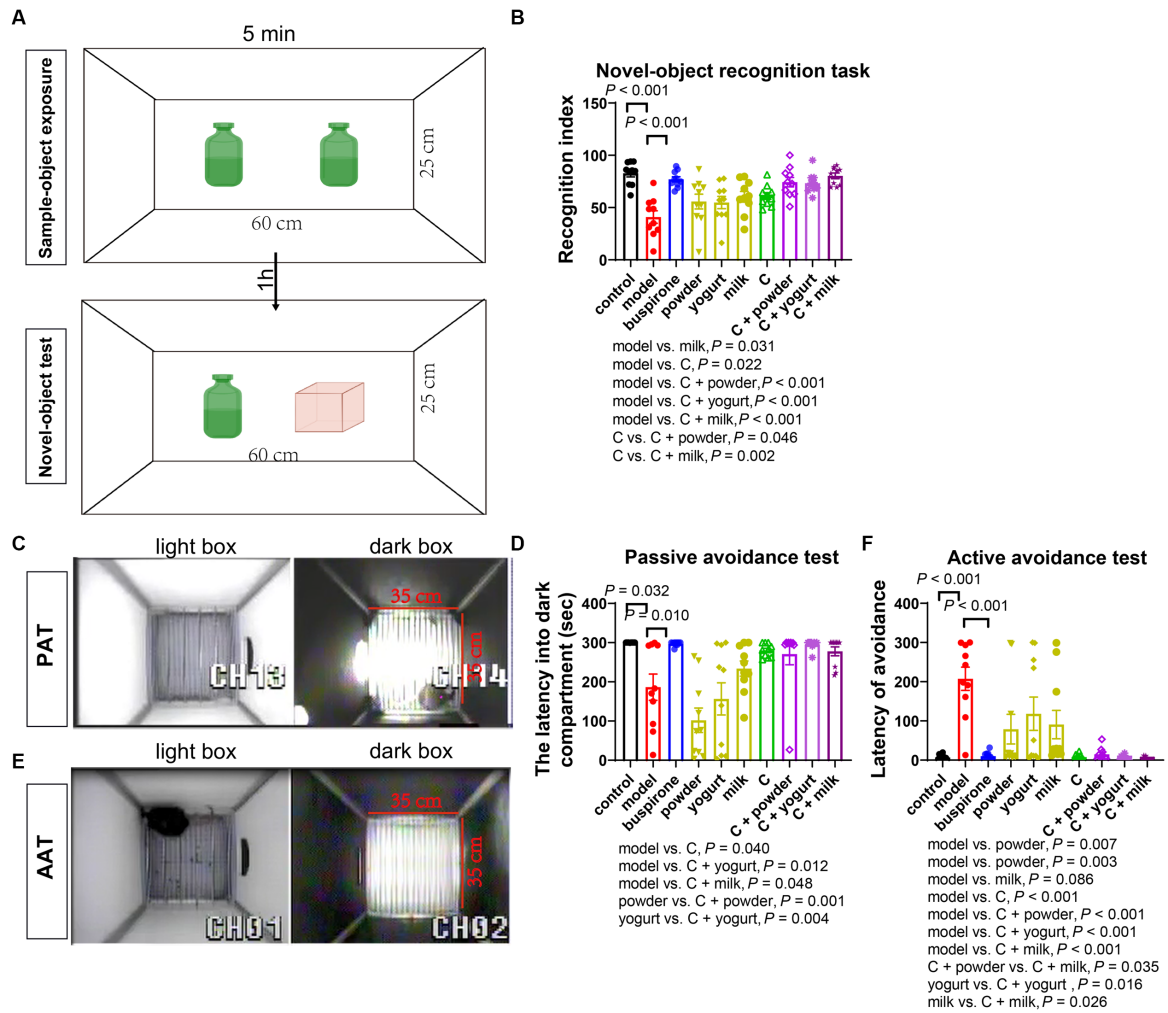
The memory improvement effects of the combination. The NOR test performed on the 32nd day. **(A)** Schematic of novel object recognition test. The recognition index analyzed by NOR in each group **(B)**. On the 36th, the avoidance test was conducted. Schematic of the PAT **(C)**. The latency to enter the dark compartment by PAT for each group on the 36th day is presented in panel **(D)**. On the 41st day; the avoidance test was conducted. Schematic of the AAT **(E)**, while the latency to avoidance by AAT for each group is shown in panel **(F)**. The data are expressed as mean + SEM with *n* = 10 in each group.

We further conducted the NOR and avoidance (PAT and AAT) tests to evaluate memory impairment in anxiety mice. On day 32, the NOR test indicated a decrease in the recognition index in the model group compared to the control (*t* = 6.071, df = 18, *p* < 0.001). This suggests that the persistent stress led to a decline in memory function. The administration of buspirone was efficacious in improving memory (Figures 4A,B).

In the PAT test, we observed a reduced latency in entering the dark box in the model and the three basal groups compared to the control (Figures 4C,D). One-way ANOVA was employed to analyze the model group and treatment groups in the present study [*F*_(8, 81)_ = 8.646, *p* < 0.001]. Additionally, the C, C + yogurt, and C + milk groups had longer latency times than the model group (*p* = 0.040, *p* = 0.012, *p* = 0.048), with the C + milk group displaying a longer latency time than the yogurt group ([powder vs. C + powder]: *p* = 0.001; [yogurt vs. C + yogurt]: *p* = 0.004; Figures 4C,D). During the AAT, a heightened latency was noted in the model group and the three basal groups upon entering the light box compared to the control (Figures 4E,F). Excluding the control group, a one-way ANOVA analysis was conducted on the remaining groups [*F*_(8, 81)_ = 8.201, *p* < 0.001]. Moreover, the C + powder group showed a more extended incubation period than the C + milk group (*p* = 0.035). For additional trajectory plots illustrating the traces of treatment groups receiving the combination, kindly consult Supplementary Figure 4.

### 3.3. WP + CP combination improved the imbalanced neurotransmitters and BDNF expression caused by anxiety

**Figure 5:**
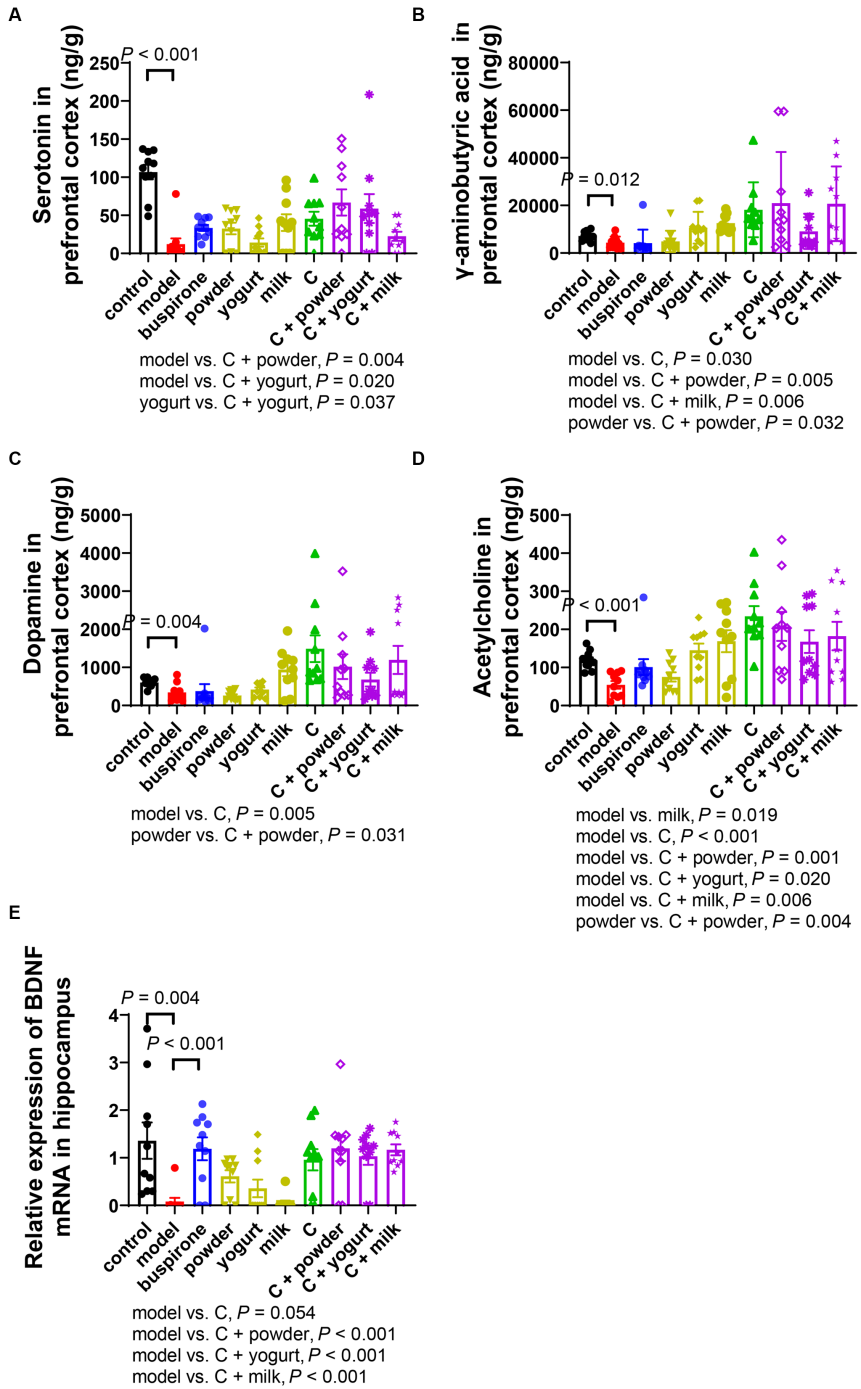
Changes of neurotransmitters in PFC and BDNF mRNA expression in the hippocampus after anxiety **(A-D)**. The concentration of serotonin (5-HT) **(A)**, γ-aminobutyric acid (GABA) **(B)**, dopamine (DA) **(C)**, and acetylcholine (ACh) **(D)** in the right prefrontal tested by LC–MS. **(E)** BDNF mRNA relative expression changes in the hippocampus. Concentrations are given in ng/g wet tissue. Data represent mean ± SEM; *n* = 10.

The occurrence and development of anxiety are closely related to changes in neurotransmitters, such as Serotonin, GABA, DA, and ACh. Serotonin and GABA are the targets of commonly used antianxiety medications. We assayed neurotransmitter concentrations in mice**’**s PFC, revealing a significant reduction in serotonin levels in the model compared to the control (*t* = 7.751, df = 18, *p* < 0.001). Excluding the control group, a one-way ANOVA analysis was conducted on the remaining groups [*F*_(8, 81)_ = 3.007, *p* < 0.001]. Notably, C + powder/yogurt induced higher serotonin levels than the model (*p* = 0.004, *p* = 0.020). In contrast, C + yogurt also caused higher serotonin concentration than yogurt (*p* = 0.037; Figure 5A). Furthermore, the model observed lower GABA concentrations than the control (*t* = 2.785, df = 18, *p* = 0.012). Excluding the control group, a one-way ANOVA analysis was conducted on the remaining groups [*F*_(8, 81)_ = 4.187, *p* < 0.001]. However, C and C + powder/milk significantly increased GABA concentrations compared to the model (*p* = 0.030, *p* = 0.005, *p* = 0.006; Figure 5B). In addition, dopamine (DA) concentration was higher in the control compared to the model (*t* = 3.270, df = 18, *p* = 0.004). Excluding the control group, a one-way ANOVA analysis was conducted on the remaining groups [*F*_(8, 81)_ = 3.648, *p* < 0.001]. The DA concentration in the C group was considerably higher than the model (*p* = 0.005; Figure 5C). Lastly, in comparison to the control group, the model group displayed a slight decrease in ACh concentration (*t* = 5.116, df = 18, *p* < 0.001). Excluding the control group, a one-way ANOVA analysis was conducted on the remaining groups [*F*_(8,81)_ = 5.284, *p* < 0.001]. Differences existed among the model and the eight treatment groups (*p* < 0.001). Moreover, the C + powder group exhibited a higher concentration of ACh when compared to the powder group (*p* = 0.004; Figure 5D).

It is widely observed that reduced BDNF protein expression is induced by chronic stress (27); we investigated the BDNF expressions in the hippocampus. BDNF expression was inhibited in the model compared to the control (*t* = 3.283, df = 18, *p* = 0.004). Excluding the control group, a one-way ANOVA analysis was conducted on the remaining groups [*F*_(8, 81)_ = 7.136, *p* < 0.001]. Conversely, buspirone treatment effectively elevated the BDNF expression (*p* < 0.001; Figure 5E). Moreover, a marked increase in the relative expression level of BDNF was observed in the C/C + yogurt/C + yogurt/C + milk group compared to the model group.

### 3.4. The correlations between behavioral tests and serotonin or BDNF expressions were highly related

**Figure 6:**
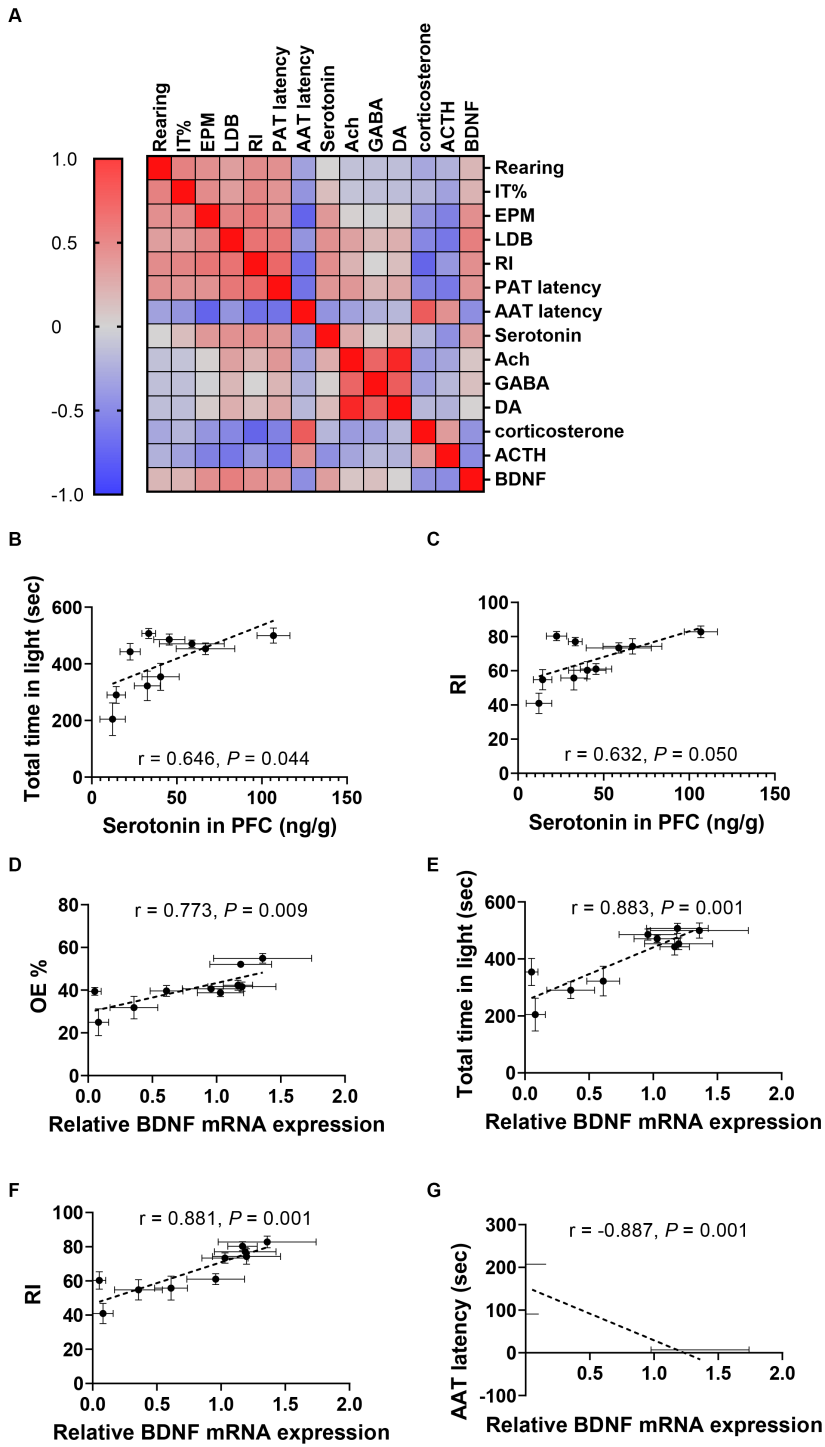
Correlations between neurotransmitters, BDNF, and anxiety-related indices. **(A)** Heatmap of the Correlation coefficient matrixes of the responses of various indicators for each mouse. The correlation coefficient matrices display a spectrum of colors, wherein intensifying shades of red correspond to heightened correlation levels while deepening shades of blue denote decreased correlation levels. **(B,C)** Correlations between 5-HT concentration in PFC and total time in light in LDB **(B)** and RI in NOR **(C)**. **(D–G)** Correlations between relative BDNF mRNA expression and OE% in EPM **(D)**, total time in light of LDB **(E)**, RI in NOR **(F)**, and AAT latency **(G)**. Data were represented as mean ± SEM, *n* = 10 in each group.

Through the comprehensive analysis of the data presented, we have verified the anxiolytic and memory-enhancing effects of WP + CP. As alterations in neurotransmitter and BDNF expression have been observed in response to anxiety, we aimed to explore the interplay among behavior, serum corticosterone, ACTH, neurotransmitters, and BDNF expression in mice (Figure 6A). Notably, a negative correlation was found between serotonin concentration in PFC and anxiety-like behaviors, such as total time in light in LDB (r = 0.646, *p* = 0.044, Figure 6B) and RI (r = 0.632, *p* = 0.050; Figure 6B). Furthermore, OE% exhibited a positive correlation with relative BDNF expression (r = 0.773, *p* = 0.009; Figure 6C), which was consistent with the correlation observed between total time in light in LDB (r = 0.883, *p* = 0.001; Figure 6D). Impressively, a strong correlation was also observed between the relative expression level of BDNF and NOR (r = 0.881, *p* = 0.001; Figure 6E) and AAT experiments (r = −0.887, *p* = 0.001; Figure 6F).

Given the observed correlation between fecal particle production and behavioral outcomes, we further investigated the potential connections between behavior, BDNF expression, and fecal excretion, as outlined in Supplementary Figure 5. Notably, a negative correlation emerged between fecal production and behaviors such as total time spent in the lit area of the LDB (r = −0.848, *p* = 0.004; Supplementary Figure 5A), AAT latency (r = 0.840, *p* = 0.005; Supplementary Figure 5B), and RI (r = −0.827, *p* = 0.006; Supplementary Figure 5C). Additionally, the number of feces positively correlated with BDNF mRNA expression relative to control levels (r = −0.958, *p* < 0.001; Supplementary Figure 5D). These correlation analysis data indicated the relatively strong reciprocity between behaviors and neurotransmitters or behaviors and BDNF expressions. The correlation matrix of behavioral and biological indicators is shown in Supplementary Table 7.

Besides neurotransmitters, numerous studies have highlighted the importance of low-grade inflammation in the pathophysiology of anxiety (28). We tested Iba1 expression, the biomarker of microglia activation, in the left hemisphere by immunohistochemistry. There were no significant differences in Iba1 microglial cell count (Supplementary Figure 6) between the control and model groups, indicating that the increase in Iba1 expression and coverage could not be ascribed to variances in the number of cells.

## 4. Discussion

The current study reported the anxiety-relieving and memory-improving effects of the WP + CP at 170 + 300 mg (human dose), both as a combination and their derived products (powder, yogurt, and milk). The dosage was designed by integrating the results from the previous human trials (29, 30), zebrafish experiments (13, 31), and the current mouse experiments. Utilizing a combined nutrient combination of WP and CP in this study is essential as it not only exhibits antianxiety and memory improvement effects, as observed with WP and CP alone, but also presents a potential cost-reduction advantage. This study investigates the rationale behind utilizing this combination and presents the necessity for further research in this area.

We employed the EOP paradigm to establish anxiety models in mice. Although foot shock stress is commonly used to establish an anxiety model, inconsistent stress induction procedures can lead to anhedonia and learned helplessness (32, 33). Therefore, we chose the EOP paradigm, which utilizes a comparatively milder physical stimulus, to construct our mouse anxiety model. This approach provides a controlled and consistent method for inducing anxiety-like behaviors in mice, which helps investigate anxiety disorders’ neurobiological basis and assess potential treatments.

In this study, we evaluated the anxiety states of mice using the OFT, EPM, and LDB tests. The results indicated that the EPM and LDB tests had more positive outcomes than the OFT test. The study confirmed the sensitivity of EPM and LDB in detecting anxiolytic compounds, proving their reliability (34). Previous research has shown that OFT is less reliable due to the administration operation**’**s interference with the actual experimental results, primarily when orally administered to mice fed different diets (35). In contrast, rearing times were the most sensitive index on OFT as it reveals a mouse**’**s exercise capacity in vertical directions (36). In our study, we observed that incorporating the combination compound C + milk yielded superior antianxiety effects compared to the milk group, as evidenced by significant differences in OFT, serum corticosterone, and ACTH indicators. These findings strongly suggest a synergistic interaction between the combined compound and milk, enhancing its potential to alleviate anxiety (37, 38).

Wang et al. and Zhao et al. investigated the neuroprotective effects of WP against memory deficits induced by lipopolysaccharide and walnut-derived peptide mechanism and pathway of mitophagy in mice (9, 39). The studies of CP on stress were started 30 years ago in animal experiments and clinical trials, showing the effects of insomnia and anxiety-improving properties (40–42). Our previous studies in zebrafish revealed that the nutrient combination of WP + CP at 56.7 + 100 μg/mL exhibited antianxiety, antioxidant, neuroprotection, and memory improvement properties (13). This is the first time to evaluate the effect of WP and CP combination. Based on dose conversion between zebrafish and mice, the current study utilized WP + CP at a dosage of 25.87 + 45.50 mg/kg, equivalent to 170 + 300 mg of the equal human dose, which proved effective in improving anxiety and memory in mice.

Studies have shown that ACTH secretion is regulated by various factors, including stress stimuli. When exposed to stress, the pituitary gland releases ACTH, which stimulates the synthesis and release of corticosterone from the adrenal cortex. Subsequently, the elevated levels of corticosterone suppress ACTH secretion through a negative feedback mechanism, maintaining the homeostasis of the endocrine system (43). The dysfunctional neurotransmitter systems exist in anxiety regulation (44). In primates, the PFC, the chief executive officer of the brain, regulates anxiety by engaging high-level regulatory strategies aimed at coping with and modifying the experience of anxiety (45). Based on these theories, we determined the concentrations of four neurotransmitters in the PFC of each mouse. In the chronic stress rodents, reduced 5-HT, GABA, and DA levels in PFC were reported (46–48), which is consistent with our model. We found that the product of combination (C + powder/yogurt) significantly increased the 5-HT concentration in PFC, demonstrating its antianxiety effects through 5-HT. However, the treated groups had even higher GABA concentrations than the control in our study. This could be due to the long-term administration of dairy products, which may promote intestinal absorption and affect immunity, the microbiome, and the gut-brain module (49–51). ACh plays a significant role in regulating muscles, the heart, the digestive tract, and the nervous system (52). Our study found that treating anxiety mice with the combinations and its products could increase ACh levels in PFC, which was in line with the memory-related behavioral tests.

Previous studies have demonstrated reduced BDNF protein expression in the hippocampus induced by chronic stress (27), consistent with our experimental results. Recent research has revealed that the amygdala, known for its role in emotions, also processes non-conditioned stimuli (53–55). This sheds new light on the intricate relationship between cognition and emotion, traditionally associated with the hippocampus (56, 57). The hippocampus is one of the key brain structures for emotional response and is particularly susceptible to endogenous stressors. Meanwhile, BDNF exerts its activity most in the hippocampus (58–60). Brain-derived neurotrophic Factor (BDNF) plays a pivotal role in the nervous system by facilitating neuronal growth, differentiation, and survival, thus serving as a critical neurotrophic factor (61). Ma et al. found that adult neurogenesis persists in the dentate gyrus of rodents and is stimulated by chronic treatment with conventional antidepressant drugs through the BDNF/Tropomyosin receptor kinase B (TrkB) signaling pathway (56).

Numerous studies have highlighted the importance of low-grade inflammation in the pathophysiology of anxiety, such as increased levels of proinflammatory cytokines in the brain (28). Inflammatory conditions promote tryptophan metabolism along the kynurenine pathway at the expense of the 5-HT pathway (62). We did not find a difference between the model and control, which may be due to the time of sampling and the brain region we chose to perform IHC assays. Additionally, activated microglia exhibited phenotypes termed M1 and M2 phenotypes. M1 microglia contribute to the development of inflammation, while M2 microglia exert anti-inflammation effects (63). We may find more precise and meaningful results if we detect M1 and M2 microglia phenotypes separately.

One of the limitations of this study was the absence of BDNF protein expression. In the following studies, we will conduct in-depth studies on the specific mechanism of WP + CP on anxiety-relieving and memory improvement, particularly the serotonin and BDNF pathways. Besides, administrating buspirone by intraperitoneal (IP) injection rather than intragastric (IG) administration might cause a slight difference in anxiety levels. Previously, we proved that IP exhibited superior therapeutic efficacy, which is more suitable for administering positive control (35). However, it did not affect the evaluation of combinations in this study. Since this study was a basic animal research project, further investigation is required to determine the effective dose of WP and CP in humans.

## 5. Conclusion

Overall, the study investigated the impact of a WP + CP combination, administered at a human dose of 170 + 300 mg, on anxiety relief and memory improvement in mice with anxiety. The combination, either alone or dissolved in products such as powder, yogurt, or milk, exhibited similar efficacy, possibly through the modulation of neurotransmitters or the BDNF pathway.

## Data availability statement

The original contributions presented in the study are included in the article/Supplementary material, further inquiries can be directed to the corresponding authors.

## Ethics statement

The animal study was approved by Institutional Animal Care and Use Committee (IACUC) of the West China Hospital, Sichuan University (Approval No. 2019194A). The study was conducted in accordance with the local legislation and institutional requirements.

## Author contributions

QL: Writing – original draft, Methodology, Validation. XJ: Validation, Writing – review & editing, Conceptualization, Resources. QZ: Validation, Project administration, Writing – review & editing. ZhZ: Writing – review & editing, Investigation, Project administration. YW: Methodology, Software, Writing – original draft. CT: Methodology, Software, Writing – original draft. BZ: Methodology, Software, Writing – original draft. HF: Resources, Writing – review & editing. JHao: Resources, Writing – review & editing. ZiZ: Resources, Writing – review & editing. JHe: Data curation, Supervision, Writing – review & editing. YZ: Conceptualization, Project administration, Writing – original draft.

## Funding

The author(s) declare that no financial support was received for the research, authorship, and/or publication of this article.

## Conflict of interest

XJ, HF, JHao, JHe, and ZiZ were employed by Inner Mongolia Yili Industrial Group Co., Ltd. and Inner Mongolia Dairy Technology Research Institute Co. Ltd.

The remaining authors declare that the research was conducted without any commercial or financial relationships constructed as a potential conflict of interest.

## Publisher’s note

All claims expressed in this article are solely those of the authors and do not necessarily represent those of their affiliated organizations, or those of the publisher, the editors and the reviewers. Any product that may be evaluated in this article, or claim that may be made by its manufacturer, is not guaranteed or endorsed by the publisher.
