# Supplementary material for: Combination of Walnut Peptide and Casein Peptide alleviates anxiety and improves memory in anxiety mices
Source: Front Nutr. 2023 Oct 6;10:1273531. doi: 10.3389/fnut.2023.1273531 (PMC10588484; doi:10.3389/fnut.2023.1273531)
Supplement: Supplementary file 1 [file Data_Sheet_1.docx]

**Combination of Walnut Peptide and Casein Peptide alleviates anxiety and improves memory in anxiety mice**

**Supplementary materials**

1. **Supplementary materials and methods**

1.1 Elevated Open Platform (EOP) and fecal particle count were measured

Expanding upon a previous study with slight modifications, we obtained images of the Elevated Open Platform (EOP). The EOP provides a highly standardized and reproducible environment that is easily constructed, allowing for partial replication of the situations and challenges faced by mice and inducing anxiety-like behavior and emotional distress. Following the elevated platform model, we recorded the fecal particles each mouse deposited on the 10 cm x 10 cm platform. This protocol was carried out over 30 days, with mice exposed to the platform for one hour per day (Supplementary Figure 1).

1.2 Assessment of animals’ physical stats

We evaluated the mice’s body weight, food intake, coat condition score, and sucrose preference test (SPT) every Tuesday from 10-12 a.m., as depicted in Figure 1. The assessment of coat condition involved evaluating seven body parts, including the head, neck, dorsal and ventral coat, tail, anterior claw, and hind claw. A score of 0 was assigned for mice with regular and soft hair, while a score of 1 was given for those with unwell-groomed and dark hair color in each part.

1.3 Immunohistochemistry

Mouse brains were first fixed in 10% buffered formalin and subsequently embedded in paraffin for immunohistochemical analysis using 4-μm sections. Our analysis utilized the anti-Iba1 antibody from Wako (Richmond, VA) at a concentration of 1:1000 to specifically detect Iba1. To quantify the number of Iba1-positive cells, we performed a meticulous count of the total number of cells that had undergone positive staining. This count was explicitly done in areas that exhibited the highest density and was performed across ten non-overlapping microscopic fields, each observed at 400× magnification in brains obtained from each experimental group of mice.

**2. Supplementary Figures**


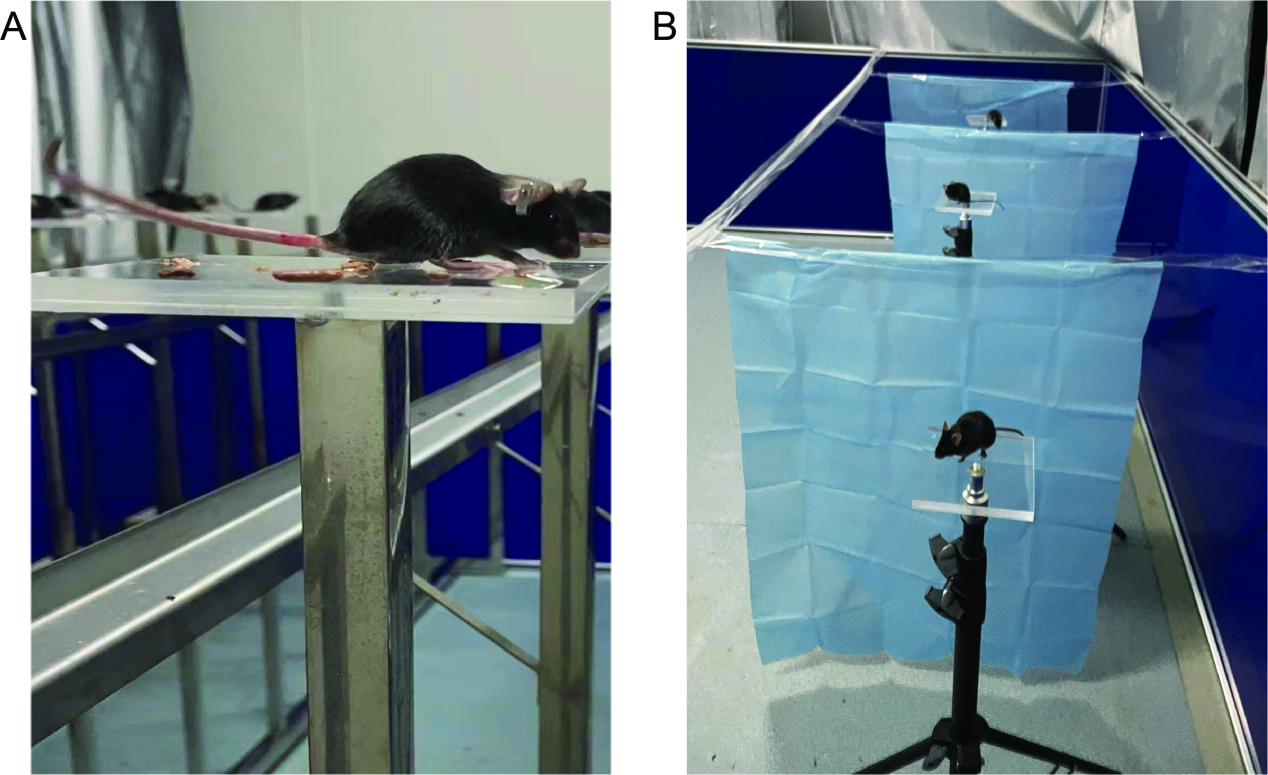


**Supplementary Figure 1. A** Elevated open platform (EOP). After modeling the elevated platform, we returned the mice to their cages and recorded the number of fecal particles dropped by each mouse on the platform. **B** During the modeling process, the mice were isolated and unable to see each other.


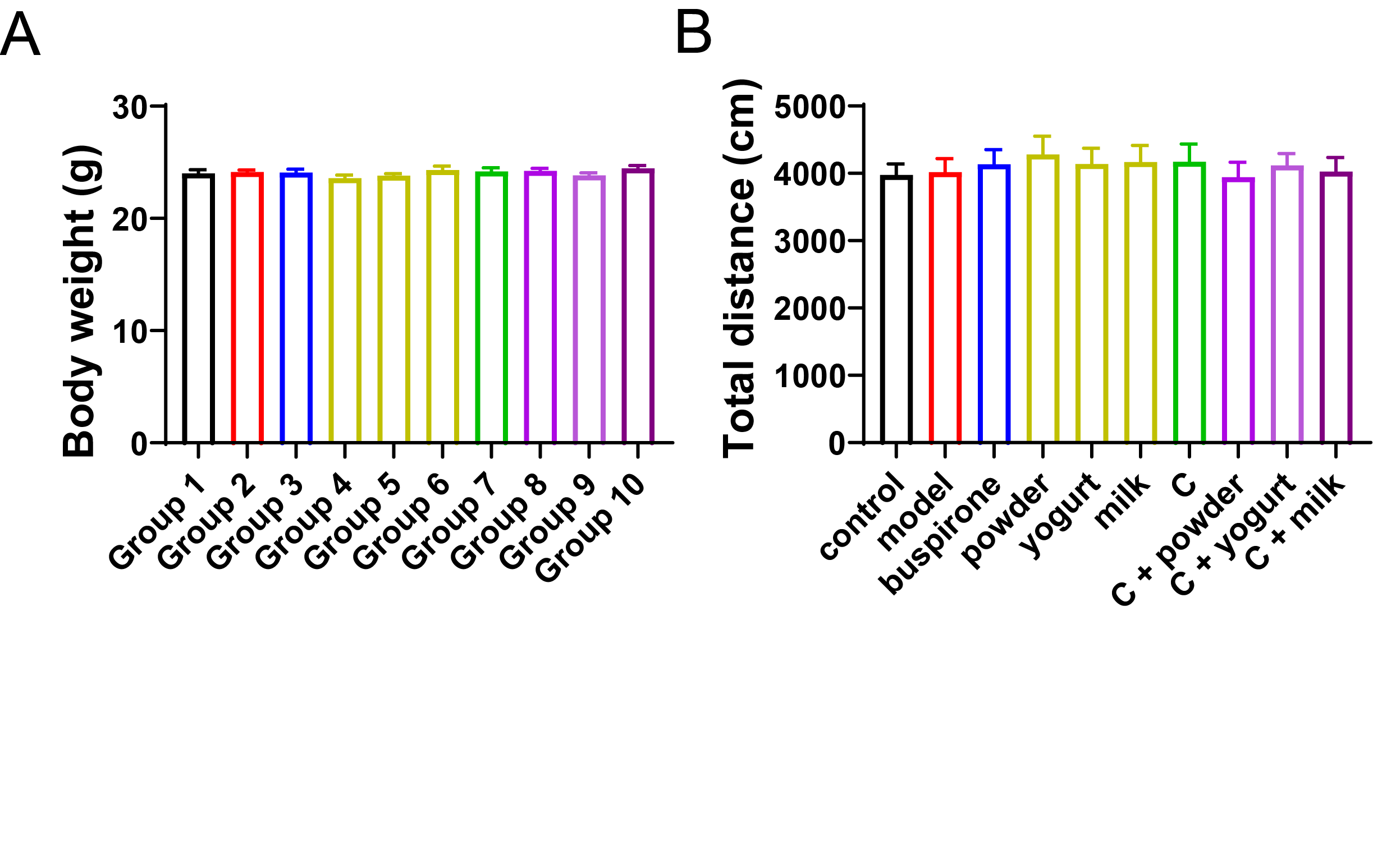


**Supplementary Figure 2. A** On the first day of the experiment, the mice were randomized based on their body weight, and no significant differences were observed between the groups. **B** After grouping the mice by weight, we evaluated their exercise capacity and found no significant differences between the groups. The data, representing mean values ± standard error of the mean (s.e.m.) for a total of 10 subjects, were analyzed using repeated measures analysis of variance (ANOVA) to investigate potential differences among groups.


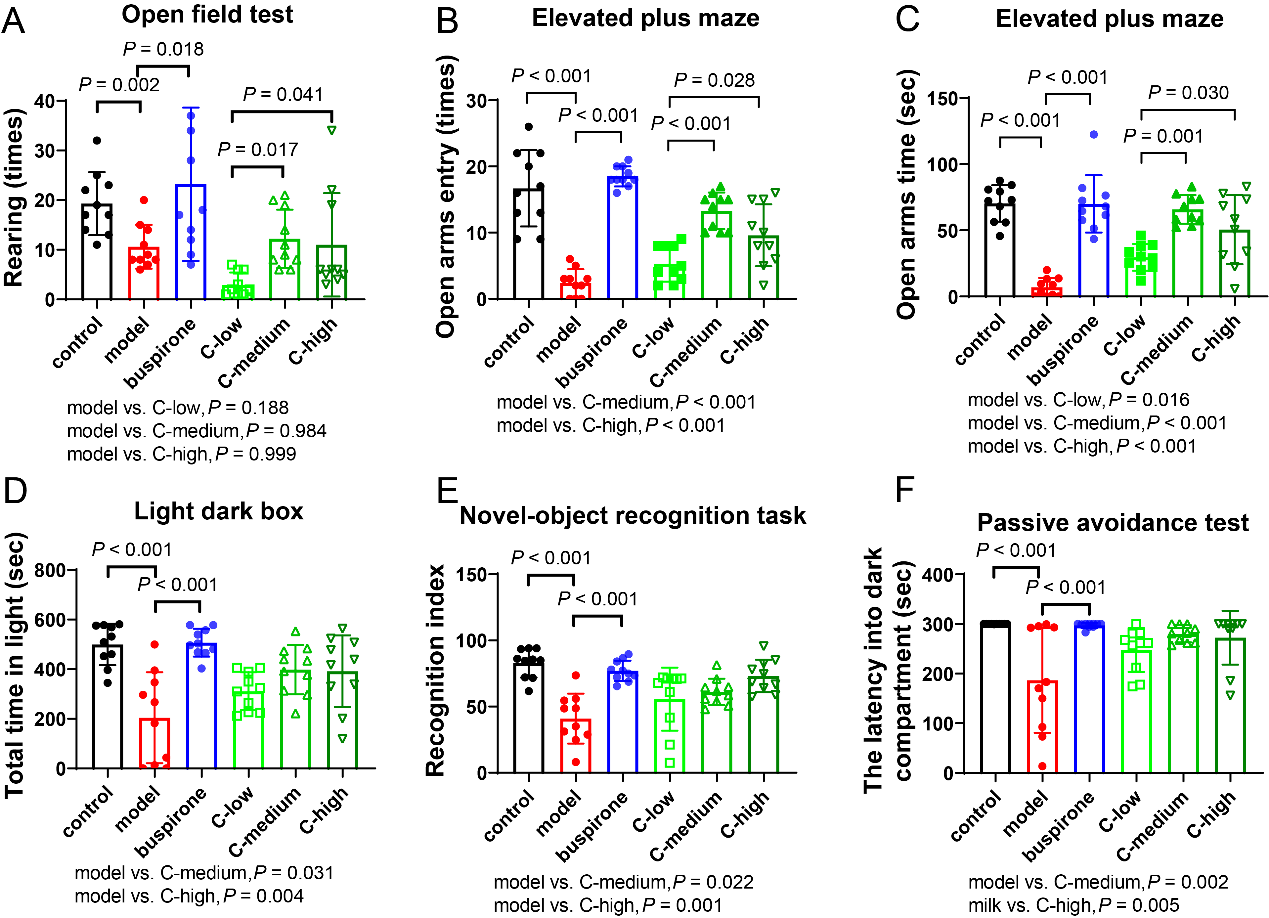


**Supplementary Figure 3.** A-B: OFT performed on the 30^th^ day. Rearing times (**A**) and changes of the percentage of inner zone time (**B**), by OFT in each group. C: EPM performed on the 32^nd^ day—changes of OE% (**C**). D LDB performed on the 35^th^ day. Total time in light (**D**). E Schematic of novel object recognition test. The recognition index analyzed by NOR in each group (**E**). On the ^3^6th, the avoidance test was conducted—the PAT (F) schematic. The data are expressed as mean ± SEM with n = 10 in each group.


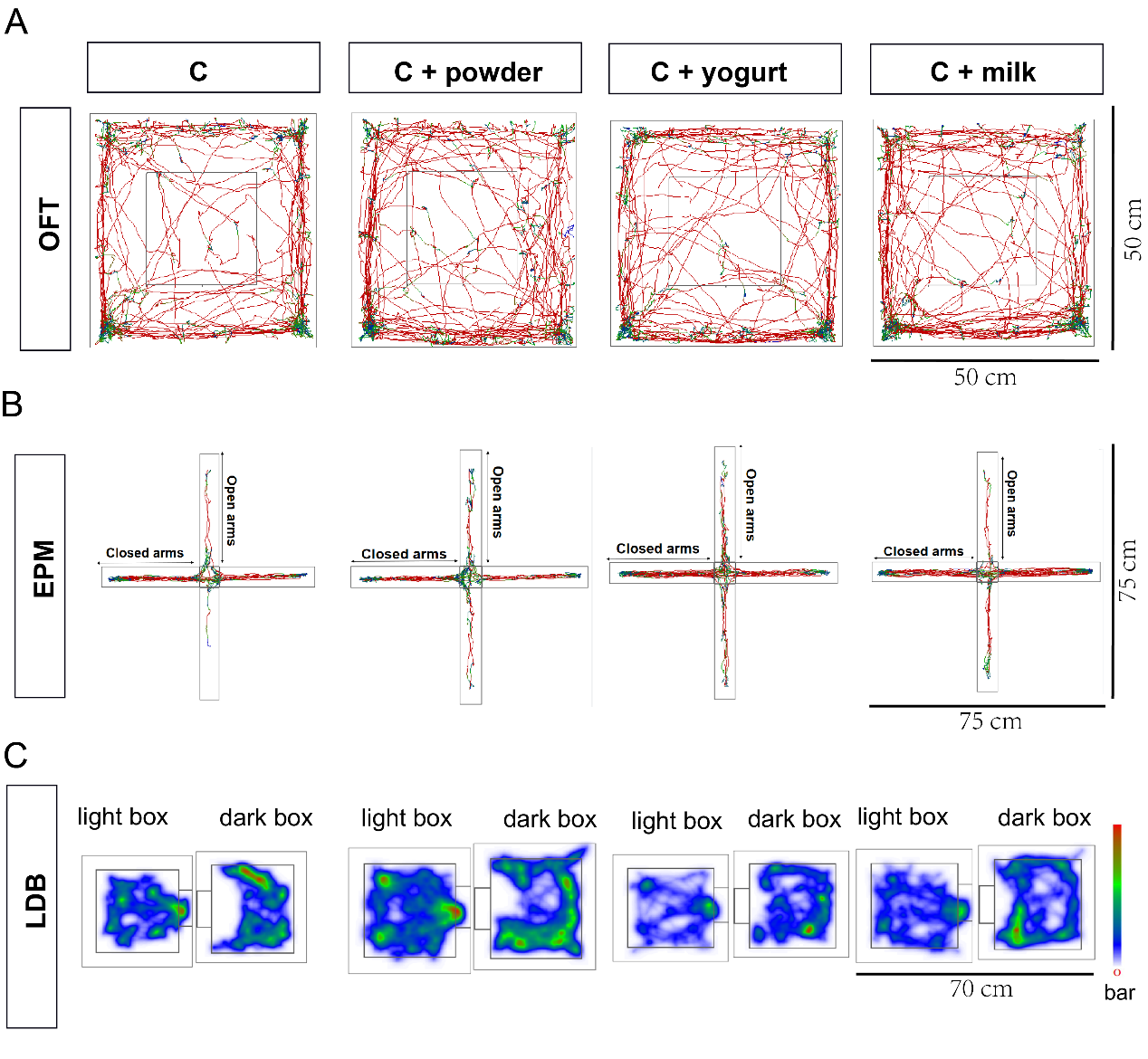


**Supplementary Figure 4.** The behavioral trace of treatment groups with combination


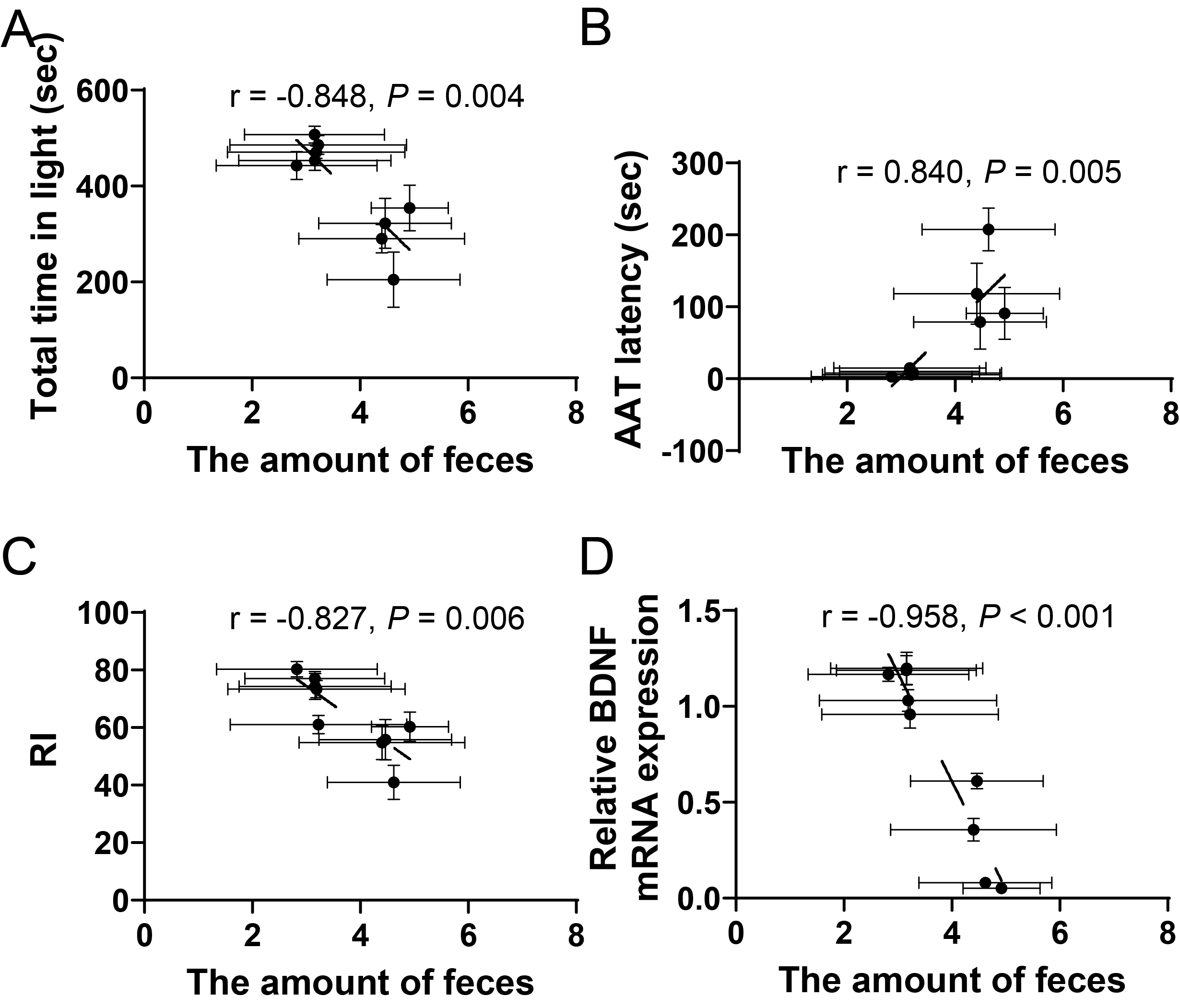


**Supplementary Figure 5** Correlations between the amount of feces and total time in light in LDB (A), AAT latency (B), recognition index in NOR test (C), and relative BDNF mRNA expression (D). Data were represented as mean ± SEM, n = 10 in each group.

**
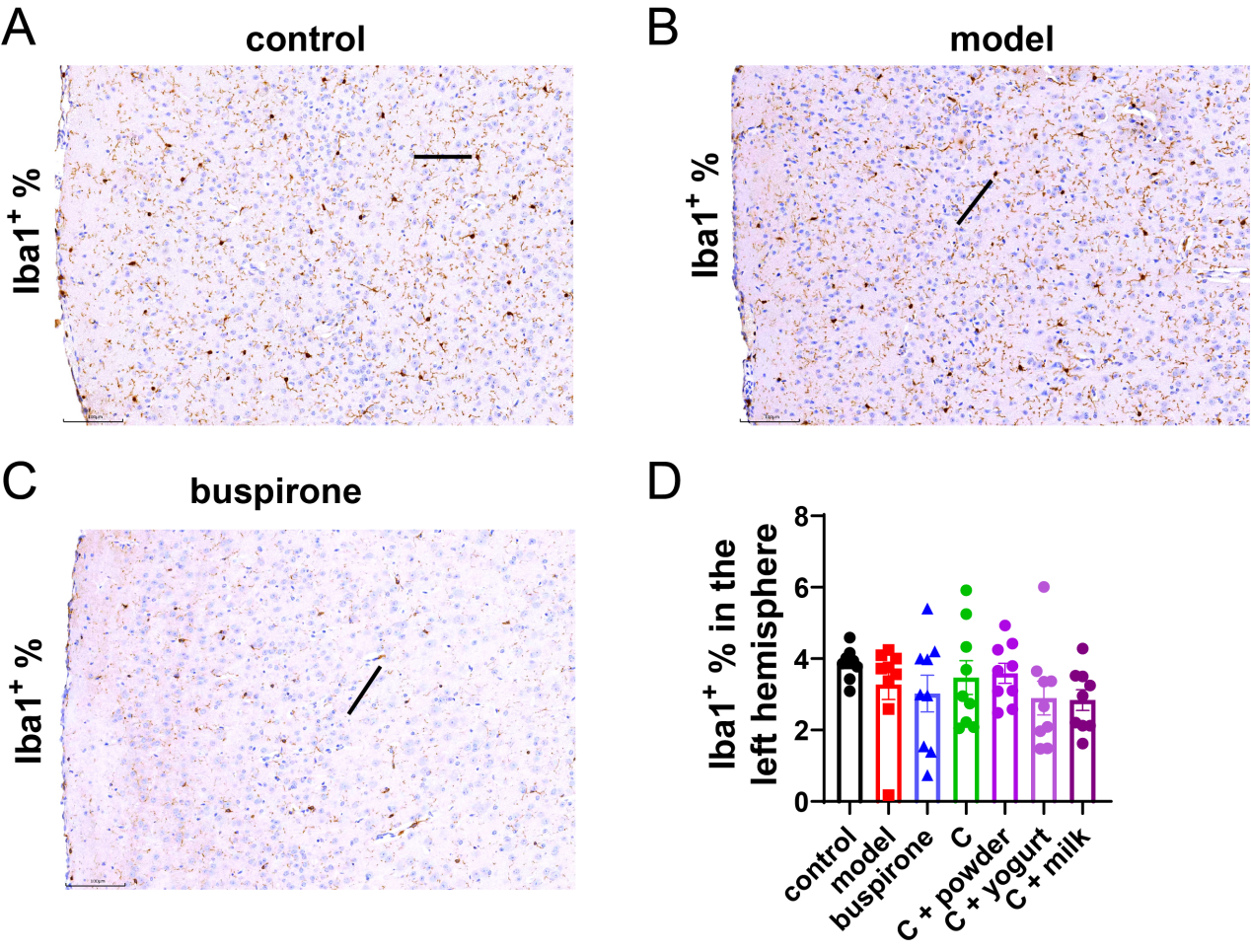
**

**Supplementary Figure 6.** Extract the left hemisphere from each group of mice and assess the marker Iba1 expression in microglial cells using immunohistochemical staining. The three representative images labeled **A**, **B** and **C** correspond to the three immunofluorescence groups. / = bushy Iba1^+^Cells **D** The variations in the percentage of astrocytes showing positive expression between different groups.

**3. Supplementary Tables**

**Supplementary Table 1A Repeated measures of body weight**

|  | **Repeated measure F-test** | | |
| --- | --- | --- | --- |
|  | **F** | **df** | ***P*** |
| **Main effects of groups** | 4.689 | 9 | **< 0.001** |
| **Main effects of days (Day 1-49)** | 21.312 | 7 | **< 0.001** |
| **Group*days** | 3.207 | 63 | **< 0.001** |

**Supplementary Table 1B The simple effect of groups in body weight (Univariate tests)**

| **Day** |  | **Sum of squares** | **df** | **Mean Square** | **F** | ***P*** |
| --- | --- | --- | --- | --- | --- | --- |
| **1** | Contrast | 14.748 | 9 | 1.639 | 0.897 | 0.531 |
|  | Error | 164.407 | 90 | 1.827 |  |  |
| **7** | Contrast | 53.772 | 9 | 5.975 | 2.917 | **0.004** |
|  | Error | 184.365 | 90 | 2.049 |  |  |
| **14** | Contrast | 18.433 | 9 | 2.048 | 1.368 | 0.214 |
|  | Error | 134.737 | 90 | 1.497 |  |  |
| **21** | Contrast | 30.723 | 9 | 3.414 | 1.566 | 0.138 |
|  | Error | 196.191 | 90 | 2.180 |  |  |
| **28** | Contrast | 131.365 | 9 | 14.596 | 5.512 | **< 0.001** |
|  | Error | 238.321 | 90 | 2.648 |  |  |
| **35** | Contrast | 181.501 | 9 | 20.167 | 10.425 | **< 0.001** |
|  | Error | 174.094 | 90 | 1.934 |  |  |
| **42** | Contrast | 157.800 | 9 | 17.533 | 7.881 | **< 0.001** |
|  | Error | 200.233 | 90 | 2.225 |  |  |
| **49** | Contrast | 234.711 | 9 | 26.079 | 12.276 | **< 0.001** |
|  | Error | 191.199 | 90 | 2.124 |  |  |

**Supplementary Table 2A Repeated measures of food intake**

|  | **Repeated measure F-test** | | |
| --- | --- | --- | --- |
|  | **F** | **df** | ***P*** |
| **Main effects of groups** | 21.188 | 9 | **< 0.001** |
| **Main effects of days (Day 1-42)** | 346.909 | 6 | **< 0.001** |
| **Group*days** | 4.906 | 54 | **< 0.001** |

**Supplementary Table 2B The simple effect of groups in food intake (Univariate tests)**

| **Day** |  | **Sum of squares** | **df** | **Mean Square** | **F** | ***P*** |
| --- | --- | --- | --- | --- | --- | --- |
| **1** | Contrast | 0.242 | 9 | 0.027 | 0.516 | 0.860 |
|  | Error | 4.701 | 90 | 0.052 |  |  |
| **7** | Contrast | 5.105 | 9 | 0.567 | 9.725 | **< 0.001** |
|  | Error | 5.249 | 90 | 0.058 |  |  |
| **14** | Contrast | 13.304 | 9 | 1.478 | 13.333 | **< 0.001** |
|  | Error | 9.978 | 90 | 0.111 |  |  |
| **21** | Contrast | 4.125 | 9 | 0.458 | 6.324 | **< 0.001** |
|  | Error | 6.522 | 90 | 0.072 |  |  |
| **28** | Contrast | 14.685 | 9 | 1.632 | 6.833 | **< 0.001** |
|  | Error | 21.492 | 90 | 0.239 |  |  |
| **35** | Contrast | 11.796 | 9 | 1.311 | 9.863 | **< 0.001** |
|  | Error | 11.960 | 90 | 0.133 |  |  |
| **42** | Contrast | 5.707 | 9 | 0.634 | 7.867 | **< 0.001** |
|  | Error | 7.255 | 90 | 0.081 |  |  |

**Supplementary Table 3A Repeated measures of coat state scores**

|  | **Repeated measure F-test** | | |
| --- | --- | --- | --- |
|  | **F** | **df** | ***P*** |
| **Main effects of groups** | 3.887 | 9 | **< 0.001** |
| **Main effects of days (Day 1-35)** | 3.304 | 5 | **0.009** |
| **Group*days** | 2.173 | 45 | **< 0.001** |

**Supplementary Table 3B The simple effect of groups in coat state scores (Univariate tests)**

| **Day** |  | **Sum of squares** | **df** | **Mean Square** | **F** | ***P*** |
| --- | --- | --- | --- | --- | --- | --- |
| **1** | Contrast | 0.090 | 9 | 0.10 | 1.000 | 0.446 |
|  | Error | 0.900 | 90 | 0.10 |  |  |
| **7** | Contrast | 1.490 | 9 | 0.166 | 2.224 | **0.027** |
|  | Error | 6.700 | 90 | 0.74 |  |  |
| **14** | Contrast | 1.440 | 9 | 0.160 | 6.000 | **< 0.001** |
|  | Error | 2.400 | 90 | 0.027 |  |  |
| **21** | Contrast | 1.800 | 9 | 0.200 | 1.184 | 0.315 |
|  | Error | 15.200 | 90 | 0.169 |  |  |
| **28** | Contrast | 2.560 | 9 | 0.284 | 3.765 | **< 0.001** |
|  | Error | 6.800 | 90 | 0.076 |  |  |
| **35** | Contrast | 3.090 | 9 | 0.343 | 2.433 | **0.016** |
|  | Error | 12.700 | 90 | 0.141 |  |  |

**Supplementary Table 4A Repeated measures of sucrose preference**

|  | **Repeated measure F-test** | | |
| --- | --- | --- | --- |
|  | **F** | **df** | ***P*** |
| **Main effects of groups** | 8.361 | 9 | **< 0.001** |
| **Main effects of days (Day 1-35)** | 101.096 | 5 | **< 0.001** |
| **Group*days** | 5.643 | 45 | **< 0.001** |

**Supplementary Table 4B The simple effect of groups in sucrose preference (Univariate tests)**

| **Day** |  | **Sum of squares** | **df** | **Mean Square** | **F** | ***P*** |
| --- | --- | --- | --- | --- | --- | --- |
| **1** | Contrast | 297.937 | 9 | 33.104 | 3.706 | **0.001** |
|  | Error | 803.593 | 90 | 8.933 |  |  |
| **7** | Contrast | 501.815 | 9 | 55.757 | 2.169 | **0.031** |
|  | Error | 2313.937 | 90 | 25.710 |  |  |
| **14** | Contrast | 1311.275 | 9 | 145.697 | 7.561 | **< 0.001** |
|  | Error | 1734.169 | 90 | 19.269 |  |  |
| **21** | Contrast | 2412.767 | 9 | 268.085 | 9.540 | **< 0.001** |
|  | Error | 2529.178 | 90 | 28.102 |  |  |
| **28** | Contrast | 1956.005 | 9 | 217.334 | 11.107 | **< 0.001** |
|  | Error | 1761.083 | 90 | 19.568 |  |  |
| **35** | Contrast | 2634.257 | 9 | 292.695 | 8.059 | **< 0.001** |
|  | Error | 3268.800 | 90 | 36.320 |  |  |

**Supplementary Table 5A Repeated measures of feces amount**

|  | **Repeated measure F-test** | | |
| --- | --- | --- | --- |
|  | **F** | **df** | ***P*** |
| **Main effects of groups** | 6.002 | 8 | **< 0.001** |
| **Main effects of days (Day 1-31)** | 27.319 | 15 | **< 0.001** |
| **Group*days** | 1.746 | 120 | **< 0.001** |

**Supplementary Table 5B The simple effect of groups in feces amount (Univariate tests)**

| **Day** |  | **Sum of squares** | **df** | **Mean Square** | **F** | ***P*** |
| --- | --- | --- | --- | --- | --- | --- |
| **1** | Contrast | 70.689 | 8 | 8.836 | 2.463 | **0.019** |
|  | Error | 290.600 | 81 | 3.588 |  |  |
| **3** | Contrast | 65.689 | 8 | 8.211 | 1.011 | 0.434 |
|  | Error | 657.600 | 81 | 8.119 |  |  |
| **5** | Contrast | 87.400 | 8 | 10.925 | 1.334 | 0.239 |
|  | Error | 663.500 | 81 | 8.191 |  |  |
| **7** | Contrast | 198.400 | 8 | 24.800 | 3.950 | **0.001** |
|  | Error | 508.500 | 81 | 6.278 |  |  |
| **9** | Contrast | 136.756 | 8 | 17.094 | 1.993 | 0.058 |
|  | Error | 694.800 | 81 | 8.578 |  |  |
| **11** | Contrast | 235.800 | 8 | 29.475 | 6.345 | **< 0.001** |
|  | Error | 376.300 | 81 | 4.646 |  |  |
| **13** | Contrast | 100.400 | 8 | 12.550 | 2.868 | **0.007** |
|  | Error | 354.500 | 81 | 4.377 |  |  |
| **15** | Contrast | 132.200 | 8 | 16.525 | 4.057 | **< 0.001** |
|  | Error | 329.900 | 81 | 4.073 |  |  |
| **17** | Contrast | 77.600 | 8 | 9.700 | 2.262 | **0.031** |
|  | Error | 347.300 | 81 | 4.288 |  |  |
| **19** | Contrast | 41.956 | 8 | 5.244 | 1.245 | 0.284 |
|  | Error | 341.200 | 81 | 4.212 |  |  |
| **21** | Contrast | 98.800 | 8 | 12.350 | 2.395 | **0.023** |
|  | Error | 417.600 | 81 | 5.156 |  |  |
| **23** | Contrast | 105.222 | 8 | 13.153 | 5.709 | **< 0.001** |
|  | Error | 186.600 | 81 | 2.304 |  |  |
| **25** | Contrast | 54.800 | 8 | 6.850 | 3.648 | **0.001** |
|  | Error | 152.100 | 81 | 1.878 |  |  |
| **27** | Contrast | 63.022 | 8 | 7.878 | 5.313 | **< 0.001** |
|  | Error | 120.100 | 81 | 1.483 |  |  |
| **29** | Contrast | 91.689 | 8 | 11.461 | 5.902 | **< 0.001** |
|  | Error | 157.300 | 81 | 1.942 |  |  |
| **31** | Contrast | 73.356 | 8 | 9.169 | 5.012 | **< 0.001** |
|  | Error | 148.200 | 81 | 1.830 |  |  |

**Supplementary Table 6 Indicators related to each group’s open field test (OFT)**

|  | **Control** | **model** | **buspirone** | **powder** | **yogurt** | **milk** | **C** | **C + powder** | **C + yogurt** | **C + milk** |
| --- | --- | --- | --- | --- | --- | --- | --- | --- | --- | --- |
| **The percentage of**  **inner zone time (IT%)** | 5.74±1.31 | 1.93±0.59 | 6.41±0.82 | 1.47±0.39 | 3.52±1.45 | 1.15±0.27 | 3.27±0.85 | 2.54±1.23 | 2.87±0.25 | 3.41±0.62 |
| **Rearing (times)** | 19.30±2.01 | 10.60±1.40 | 23.20±4.88 | 7.00±1.48 | 7.50±1.50 | 6.80±2.26 | 12.2±1.86 | 9.90±2.47 | 18.2±2.47 | 15.7±2.10 |
| **Entries in Center (times)** | 16.00±2.67 | 7.80±1.37 | 19.20±2.53 | 6.50±1.72 | 7.20±1.53 | 4.30±1.03 | 11.60±2.99 | 6.10±1.63 | 14.3±1.21 | 12.4±2.36 |
| **Total distance (cm)** | 2159±213.5 | 1737±198.9 | 3254±264.4 | 1601±251.2 | 1632±183.9 | 1261±109.6 | 1626±116.2 | 1208±98.39 | 2878±309.8 | 2769±153.2 |

|  | **rearing** | **IT%** | **OE%** | **Total time in light (sec)** | **RI** | **PAT latency (sec)** | **AAT latency (sec)** |
| --- | --- | --- | --- | --- | --- | --- | --- |
| **Serotonin in PFC**  **(ng/g)** | r = 0.002 | r = 0.107 | r = 0.274** | r = 0.317** | r = 0.334** | r = 0.284** | r = - 0.307** |
|  | *P* = 0.981 | *P* = 0.289 | *P* = 0.006 | *P* = 0.001 | *P* = 0.001 | *P* = 0.004 | *P* = 0.002 |
| **ACh in PFC**  **(ng/g)** | r = -0.091 | r = - 0.084 | r = 0.018 | r = 0.232* | r = 0.155 | r = 0.279** | r = - 0.238* |
|  | *P* = 0.366 | *P* = 0.409 | *P* = 0.86 | *P* = 0.02 | *P* = 0.123 | *P* = 0.005 | *P* = 0.017 |
| **GABA in PFC**  **(ng/g)** | r = -0.102 | r = - 0.102 | r = - 0.02 | r = 0.133 | r = 0.001 | r = 0.145 | r = - 0.17 |
|  | *P* = 0.314 | *P* = 0.314 | *P* = 0.845 | *P* = 0.186 | *P* = 0.99 | *P* = 0.15 | *P* = 0.091 |
| **DA in PFC**  **(ng/g)** | r = -0.107 | r = - 0.112 | r = 0.041 | r = 0.168 | r = 0.099 | r = 0.202* | r = - 0.137 |
|  | *P* = 0.29 | *P* = 0.269 | *P* = 0.686 | *P* = 0.096 | *P* = 0.325 | *P* = 0.044 | *P* = 0.173 |
| **Serum Corticosterone (ng/mL)** | r = -.212* | r = - 0.156 | r = - 0.294** | r = - 0.372** | r = - 0.530** | r = - 0.395** | r = 0.572** |
|  | *P* = 0.034 | *P* = 0.122 | *P* = 0.003 | *P* < 0.001 | *P* < 0.001 | *P* < 0.001 | *P* < 0.001 |
| **Serum ACTH (pg/mL)** | r = -0.169 | r = - 0.237* | r = - 0.393** | r = - 0.444** | r = - 0.285** | r = - 0.443** | r = 0.321** |
|  | *P* = 0.093 | *P* = 0.018 | *P* < 0.001 | *P* < 0.001 | *P* = 0.004 | *P* < 0.001 | *P* = 0.001 |
| **Relative expression of BDNF**  **mRNA in hippocampus** | r = 0.137 | r = 0.153 | r = 0.327** | r = 0.404** | r = 0.327** | r = 0.309** | r = - 0.335** |
|  | *P* = 0.175 | *P* = 0.128 | *P* = 0.001 | *P* < 0.001 | *P* = 0.001 | *P* = 0.002 | *P* = 0.001 |

**Supplementary Table 7 Correlation matrix of behavioral and biological indicators**

** The correlation is significant at the 0.01 level (two-tailed).

* The correlation is significant at the 0.05 level (two-tailed).
